# Tris(pentafluorophenyl)borane-catalyzed Hydride Transfer Reactions in Polysiloxane Chemistry—Piers–Rubinsztajn Reaction and Related Processes

**DOI:** 10.3390/molecules28165941

**Published:** 2023-08-08

**Authors:** Slawomir Rubinsztajn, Julian Chojnowski, Urszula Mizerska

**Affiliations:** Centre of Molecular and Macromolecular Studies of Polish Academy of Sciences, Sienkiewicza 112, 90-636 Lodz, Poland; urszula.mizerska@cbmm.lodz.pl

**Keywords:** tris(pentafluorophenyl)borane, Piers–Rubinsztajn reaction, mechanism, dehydrocarbonative condensation, dehydrogenative condensation, hydride transfer polymerization

## Abstract

Tris(pentafluorophenyl)borane (TPFPB) is a unique Lewis acid that catalyzes the condensation between hydrosilanes (Si-H) and alkoxysilanes (Si-OR), leading to the formation of siloxane bonds (Si-OSi) with the release of hydrocarbon (R-H) as a byproduct—the so-called Piers–Rubinsztajn reaction. The analogous reactions of hydrosilanes with silanols (Si-OH), alcohols (R-OH), ethers (R-OR′) or water in the presence of TPFPB leads to the formation of a siloxane bond, alkoxysilane (Si-OR or Si-OR′) or silanol (Si-OH), respectively. The above processes, often referred to as Piers–Rubinsztajn reactions, provide new synthetic tools for the controlled synthesis of siloxane materials under mild conditions with high yields. The common feature of these reactions is the TPFPB-mediated hydride transfer from silicon to carbon or hydrogen. This review presents a summary of 20 years of research efforts related to this field, with a focus on new synthetic methodologies leading to numerous previously difficult to synthesize well-defined siloxane oligomers, polymers and copolymers of a complex structure and potential applications of these new materials. In addition, the mechanistic aspects of the recently discovered reactions involving hydride transfer from silicon to silicon are discussed in more detail.

## 1. Introduction

**Figure 1:**
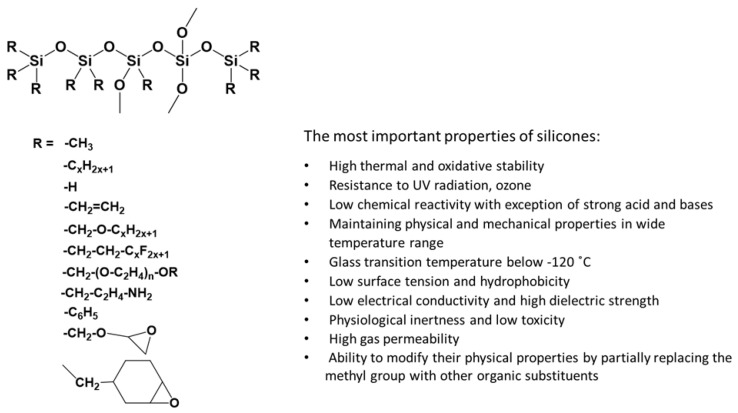
General structure of silicones and the most important properties of these materials.

Polysiloxanes, commonly known as silicones, are a class of synthetic polymeric materials containing Si linked to oxygen in the main chain. They can be linear, branched or cross-linked macromolecules. The Si atom is additionally linked by a Si-C bond with organic substituents such as methyl, phenyl, ethyl and others (Figure 1). The most common are polydimethylsiloxanes, whose backbone is built of SiMe_2_O units. They have a set of unique properties, which are superior to typical organic polymers.

These properties make silicones the material of choice for a wide range of commercial applications, including transportation, construction, electronics, healthcare, industrial processes, personal care and consumer products, and more [1]. The estimated amount of silicone materials produced in 2023 should reach over 2860 kilotons [2].

The first synthesis of siloxane material was reported by Kipping at the beginning of the 20th century [3]. The commercial production of these materials began in the late 1930s in the USA by two companies, Corning Glass Works and General Electric Company, which were looking for new insulation materials. For anyone interested in the historical development of the silicone industry, we recommend Herman Liebhafsky’s book [4].

**Scheme 1:**
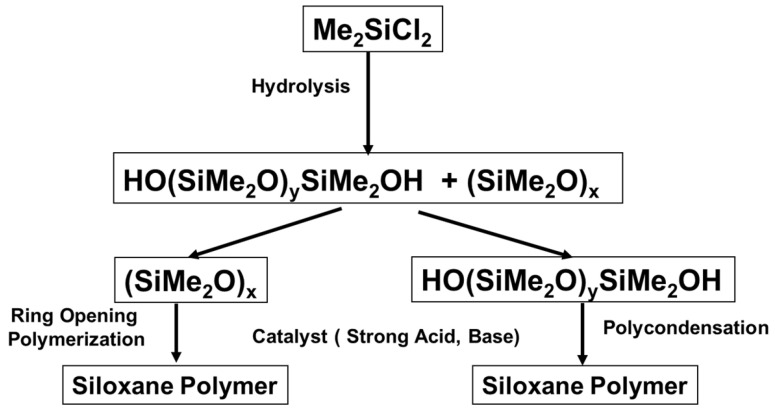
Industrially practiced processes to manufacture siloxane polymers.

Currently, silicone materials are mostly produced from dimethyldichlorosilane, Me_2_SiCl_2_, by two general processes: the polycondensation of short linear siloxanols and ring-opening polymerization of cyclic monomers such as octamethylcyclotetrasiloxane (D_4_) in the presence of a strong acid or bases [1] (Scheme 1).

Due to the high reactivity of siloxane bonds in the presence of strong acids or bases, the above processes are not selective and generally lead to the formation of silicone materials with random structures, which makes it very difficult to synthesize well-defined polymers and copolymers.

There are many other condensation reactions leading to the formation of siloxane bonds that have been discovered in the last 80 years and are practiced in the silicone industry [1,5] (Table 1). The condensation of SiH + HOSi is also promoted by heavy-metal complexes such as Pt, Pd, Rh and Ir, and was reported by Kawakami et al. [6,7] and Mark [8]. However, most currently known condensation reactions do not have the desired selectivity; are not devoid of side reactions; use relatively large amounts of catalysts, including strong acids, bases or organometallic compounds of various metals such as tin, titanium, platinum and others; and produce difficult to remove byproducts that can affect the long-term stability of the final silicone products.

For these reasons, it is highly desirable to develop new synthetic methods that would enable the production of silicone materials with a well-controlled structure that are needed for new applications. This demand is met by the dehydrocarbonative condensation between hydrosilanes and alkoxysilanes catalyzed by TPFPB, leading to the formation of a siloxane bond, which is a subject of this review.

**Figure 2:**
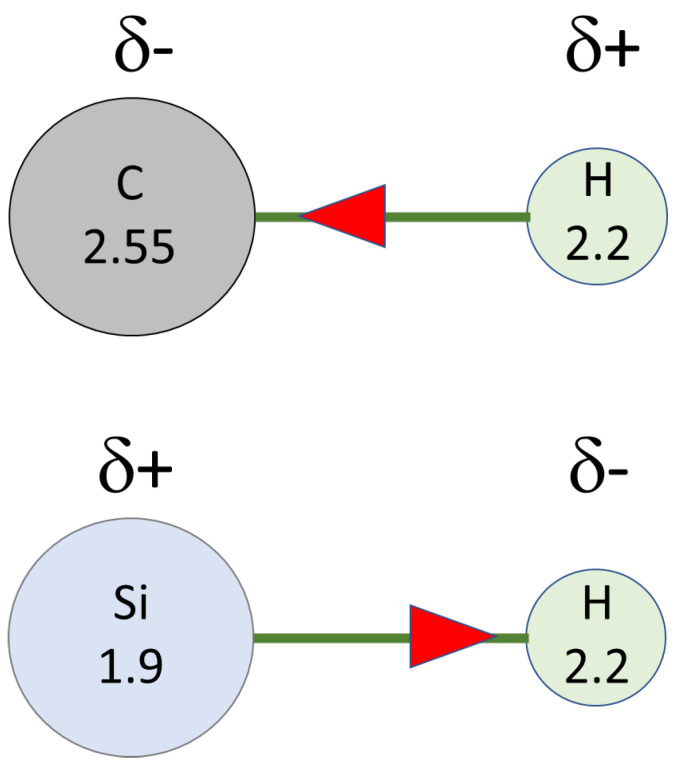
Polarization of C-H and Si-H bonds.

As mentioned earlier, the backbone of silicone materials consists of silicon and oxygen atoms (Figure 1). Silicon is the neighbor of carbon in the same XIV group in the periodic table, which is the reason for many similarities of these elements. However, silicon is more electropositive than carbon. According to the Pauling scale, silicon has an electronegativity (χ) of 1.90, while carbon has a χ of 2.55. Between them is hydrogen with a χ of 2.20. This means that the Si-H bond is polarized in the opposite direction of the C-H bond (Figure 2). Therefore, a Lewis acid can pick up hydrogen as a hydride ion from silicon.

Tris(pentafluorophenyl)borane, B(C_6_F_5_)_3_ (TPFPB), was synthetized by Massey and Park in 1964 [9]. It has some unique properties that were not fully recognized until the 1990s. It is a Lewis acid that is strong enough to pull the hydrogen with an electron away from the silicon, forming the unstable anion (C_6_F_5_)_3_BH^−^. This anion easily transfers the H^−^ to another electrophilic center, which may be a carbon atom. Hydride cleavage from silicon mediated by TPFPB occurs in many processes that are important in organic and organosilicon chemistry. It can be considered as a key stage of the silylation and reduction of organic compounds with various reactants having the SiH group in the presence of catalytic amounts of TPFPB [10]. Many reactions of SiH/TPFPB reagents that are important in organic chemistry begin with SiH bond cleavage promoted by TPFPB. See review articles [11,12,13]. SiH/TPFPB mixtures reduce alcohols, ethers and carbonyl compounds such as aldehydes, ketones, carboxylic acids and their derivatives [14,15,16,17,18]. The full reduction of carbonyl compounds to hydrocarbons proceeds smoothly in the presence of excess SiH compounds. Piers et al. reported that alcohols are converted to silyl ethers and the further reduction of silyl ethers to alkanes occurs with TPFPB-mediated hydride transfer [17]. Rubinsztajn et al. recognized that the same reaction that produces disiloxane as a byproduct of the reduction processes can be used to form siloxane and silyl ether polymers [19,20,21,22]. This process was named the Pierce–Rubinsztajn (P-R) reaction [23] and found numerous applications in the synthesis of siloxane polymers. See recent papers [24,25,26,27]. The P-R reaction allows the synthesis of many siloxanes and silyl ether polymers and their copolymers. In this process, the hydride ion, H^−^, is cleaved from silicon by TPFPB and transferred to carbon to form a siloxane or silyl ether bond together with the formation of a hydrocarbon, which is usually a volatile compound such as methane or ethane and can be easily removed from the reaction mixture (Table 2, Rows 1 and 2).

In similar reactions of hydrosilanes (SiH) with various silanols (Table 2, Row 3), alcohols (Table 2, Row 4) or water (Table 2, Row 5), TPFPB catalyzes the transfer of the hydride ion from the Si-H reagent to hydrogen instead of carbon. These reactions, which are often called Piers–Rubinsztajn reactions, create new Si-OSi, Si-OC or Si-OH bonds, respectively, but the byproduct of these reactions is hydrogen. Other processes are the TPFPB-mediated hydride exchange between two silicon atoms, which leads to a metathetic exchange of functional groups between two silicon compounds (Table 2, Rows 6, 7, 8 and 9). The main feature of all these reactions (Table 2) is the hydride transfer from the silicon atom to another atom catalyzed by TPFBP. The reactions (Table 2, Rows 3–9) are closely related to the P-R reaction, and they will also be discussed in this review.

Some other reactions of SiH reagents promoted by TPFPB are also related to P-R reactions. Zhang and Brook showed that pentamethyldisiloxane can break the organodisulfide bridges in silicone elastomers with TPFPB as a catalyst [28]. Rubinsztajn used the same catalyst to react diphenylsilane with trimethyl borate and make a highly branched borosiloxane resin with SiOB bonds [29]. Drozdov et al. followed this method to cross-link SiH-containing polysiloxanes with di- or trifunctional alkyl borates and produced various polyborosiloxane materials [30].

Although TPFPB has been a unique catalyst for P-R reactions, other catalysts have recently been proposed for analogous reactions. Fritz-Langhal reported the synthesis of the pentamethylcyclopentadienyl silicon (II) cation, Cp*Si:^+^, and its German (II) analogue. These compounds may be even more active catalysts for Piers-Rubinsztajn reactions than TPFPB [31,32,33]. Körte and co-workers made tris(perfluorotolyl)borane, which is a stronger Lewis acid than TPFPB and could also catalyze P-R reactions [34]. This review only covers the reactions catalyzed by TPFPB.

## 2. Mechanism of Piers–Rubinsztajn Reaction

**Scheme 2:**
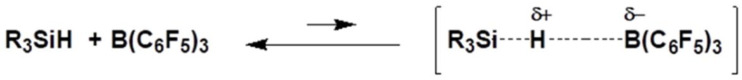
Interaction of the Si-H reagent with TPFBP.

The Piers–Rubinsztajn reaction has a similar mechanism to the reduction and silylation of organic compounds [14]. It is therefore instructive to show the mechanistic features of these previously studied reactions, such as the reduction of carbonyl compounds by a SiH-containing reagent catalyzed by TPFPB. Extensive spectroscopic and computational evidence supported by kinetic studies showed that the first step in these reactions is the formation of an adduct of the Si-H reagent with the borane (Scheme 2) [10,11,35].

**Figure 3:**
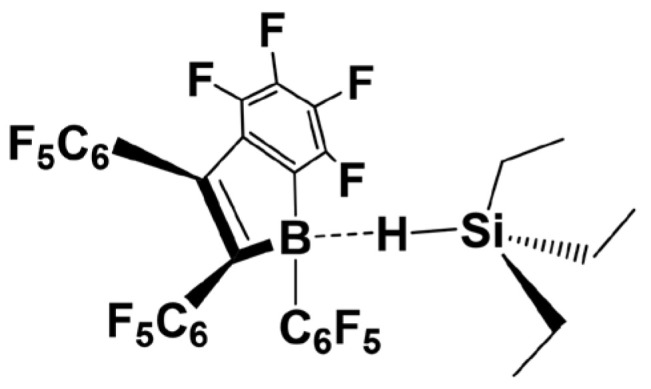
Isolated adduct of 1,2,3-tris(pentafluorophenyl)-4,5,6,7-tetrafluoro-1-boraindene with Et_3_SiH.

It was not clear if this adduct was an encounter complex or a diffusionally equilibrated species. Piers and co-workers shed some light on this question. They synthesized an antiaromatic fluoroborane of a special structure, i.e., 1,2,3-tris(pentafluorophenyl)-4,5,6,7-tetrafluoro-1-boraindene, which is a much stronger Lewis acid than TPFPB. They isolated and characterized the hydrosilane–borane complex (Figure 3) [36].

A similar complex formation is assumed to be the first step of the carbonyl reduction reaction. It is also accepted that this complex appears in the initial step of the Piers–Rubinsztajn reactions, which is supported by computational studies [37]. It should be mentioned that TPFPB is perhaps unique among the common Lewis acids and is able to form the hydrosilane–borane complex in competition with the complexation of the borane with an uncharged oxygen nucleophile, such as siloxane, ether, a carbonyl compound and others, present in the reaction mixture. TPFPB having acidity comparable to BF_3_ and AlCl_3_ [38] is much softer than these acids due to the less ionic character of the B-C bond and delocalization of the electron density from the p-fluorine atom to boron due to the propeller-like arrangement of its pentafluorophenyl groups [34]. This softness of TPFPB is the reason why it is susceptible to interactions with the soft Si-H base and soft hydride ion [39].

**Scheme 3:**
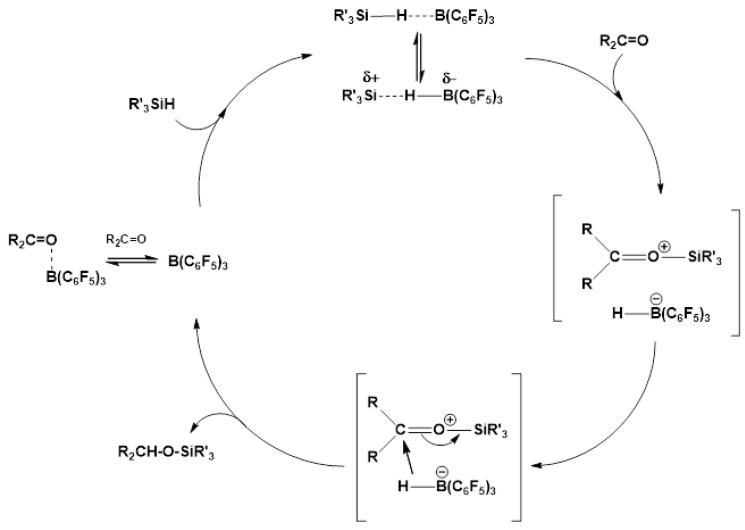
The Piers mechanism of carbonyl compound reduction (hydrosilylation) catalyzed by TPFPB.

The common mechanism of carbonyl reduction assumes that the positive silicon in the borane-HSi complex is attacked by a nucleophile like a carbonyl (Scheme 3). This idea is backed up by studies using stereogenic silane, which proved that the reaction is a bimolecular synchronous substitution of the SN2 type at silicon [40].

**Scheme 4:**
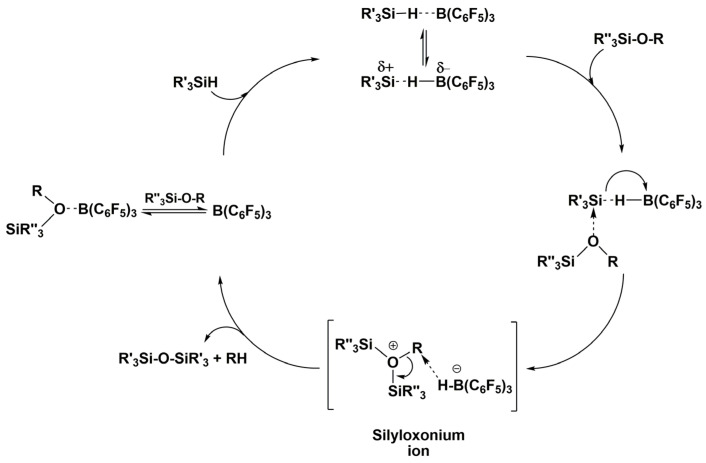
Mechanism of the Piers–Rubinsztajn reaction.

Results of kinetic and spectroscopic studies of the P-R reaction are in agreement with the formation of a silyloxonium ion as an intermediate (Scheme 4) [37]. It was confirmed with theoretical studies [35] and combined experimental and computational studies [41]. This intermediate salt decomposes with hydride transfer from the borane anion to carbon to produce disiloxane as the P-R reaction product and hydrocarbon. Hydride transfer to another silicon is also possible and leads to functional-group exchange products (Table 2, Rows 6–9), as Section 7 explains. When both reagents had the same three organic substituents R on silicon, metathesis was not visible. The formation of the disiloxane product that could be observed allowed the rate of the P-R reaction to be determined [37].

Nucleophilic substitution on the silicon concerting with the hydride transfer to carbon cannot be ruled out. However, computational studies showed the minimum of free enthalpy on the reaction pathway for the siloxonium ion [42]. The existence of the silyloxonium ion was proved experimentally [42,43]. The HB(C_6_F_5_)_3_^−^ counter-ion is a known species; see, for example, [44].

**Figure 4:**
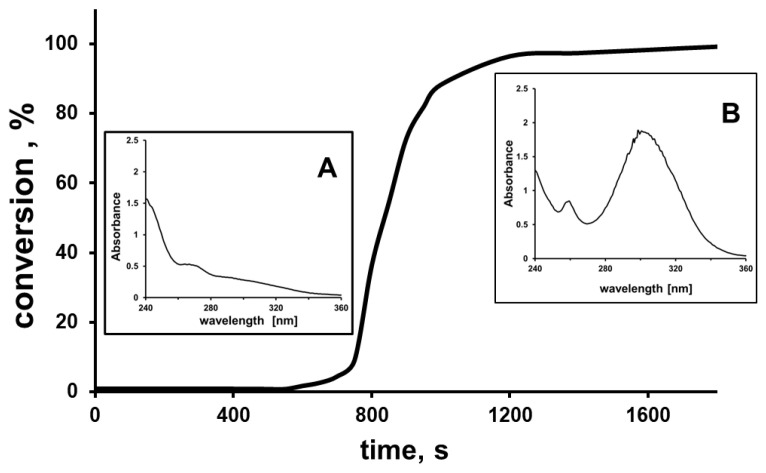
Conversion–time plot for the reaction of Ph_2_MeSiH with Ph_2_MeSin-Oct in the presence of TPFPB. (**A**)—UV spectra of the reaction mixture before the reaction started; (**B**)—UV spectra of the reaction mixture after the reaction started.

It is generally accepted that TPFPB is not easily hydrolyzed, but the presence of water affects its reactivity. The interaction of this borane with water has been extensively studied; see [45,46] and the references included there. TPFPB forms strong complexes with one, two or three water molecules. In solvents that are not nucleophilic like toluene, TPFPB can bond with more water molecules through H-bonding. (C_6_F_5_)_3_B∙H_2_O is a strong Brönsted acid with a similar strength to HCl [45]. It was recently reported that the TPFPB∙H_2_O complex is an effective catalyst of the P-R reaction [47]. Commercially available TPFPB used without thorough purification, when dissolved in a hydrocarbon solvent, usually appears as a water adduct, which is the reason for the induction period in P-R reactions [37,48]. The water bound to the borane can be removed by reacting with the SiH reagent to form a disiloxane and hydrogen. Pure TPFPB shows a strong charge transfer band in the UV spectrum with a maximum at about 310 nm (Figure 4). This band does not exist in the presence of water [37]. The interaction of borane with water causes the induction period that is often observed in the P-R reaction, as Figure 4 shows.

The induction period in the P-R reaction was not observed when water-free TPFPB was added to the solution of alkoxysilane and hydrosilane, even containing a small amount of water. This means that water cannot compete well with the complexation of the SiH compound by borane under these conditions [48]. In studies of the hydride transfer ring-opening polymerization of hexamethylcyclotrisiloxane, D_3_, in toluene initiated with PhMe_2_SiH in the presence of TPFPB, an induction period was observed after adding the catalyst to the solution of the monomer and initiator. Ganachaud et al. reported that a condensation of dimethyldimethoxysilane with 1,1,3,3-tetramethyldisiloxane in an aqueous emulsion in the presence of TPFPB yields a high-molecular-weight polysiloxane polymer [49,50]. This reaction did not occur with HCl, which seems to show that it was not catalyzed by a Brönsted acid. A complex process with three TPFPB-catalyzed condensation reactions, SiH + SiOMe, SiH + SiOH and SiOH + SiOH, leading to the formation of a siloxane bond, was suggested.

In any case, the hydride transfer mechanism for P-R reactions in the presence of water should be elucidated. TPFPB, being a Lewis acid comparable in strength to BF_3_ or slightly stronger [51], forms complexes with uncharged weak oxygen nucleophiles, such as diethyl ether and THF [52,53]. It also makes mixed complexes with water and weak nucleophiles [54]. These complexes might dissociate more easily to release the borane than the complex with water alone.

B(C_6_F_5_)_3_ is often considered to be stable under P-R conditions. However, a decrease in its activity was observed, especially in the case of slower P-R reactions and a low TPFPB concentration. TPFPB reacts with the SiH reagent, substituting one C_6_F_5_^-^ group on silicon, forming HB(C_6_F_5_)_2_ that is catalytically inactive in P-R reactions [55]. Kinetic studies of this reaction showed that its rate depends on organic groups bonded to silicon [56].

## 3. Hydride Transfer from Si to C: Piers–Rubinsztajn Reaction Leading to SiOSi

Recently, interest in Piers–Rubinsztajn and related reactions has increased because they allow for the efficient synthesis of siloxane polymers with various and sometimes unique structures under mild conditions and without using heavy-metal catalysts. These synthetic methods were summarized in recent review papers [26,27] and some earlier knowledge was incorporated into book chapters [23,50]. Practical aspects of the P-R reactions with emphasis on their applications were discussed in papers [25,26] and the paper by Wakabayashi and Kuroda focused on the use of the P-R reaction in the non-solvent sol-gel technique [57].

There are many methods for the synthesis of polysiloxanes, but many of them are difficult to control and require the use of heavy-metal catalysts, which are difficult to remove from the polymer and may deteriorate the properties of the final products. Some syntheses require a high temperature, and a large amount of undesired cyclic oligomers are usually formed in these reactions. It is difficult to obtain copolymers with regular chain microstructures. The P-R reaction provides new synthetic methods to overcome these problems. TPFPB is a very efficient catalyst operating at an ambient temperature and produces a volatile hydrocarbon as the condensation byproduct. The P-R condensation is often used to obtain polymer chain structures that are not available or difficult to obtain with other methods. The P-R reaction is often a unique method for the synthesis of polysiloxanes with a high degree of control of the macromolecule microstructure. In the following subsections, the current developments in this field will be presented.

### 3.1. Cross-Linking of Linear Polysiloxanes in the Preparation of Silicone Elastomers, Coatings and Foams

The use of the P-R reaction for cross-linking siloxane polymers with SiH functional groups was first described by Rubinsztajn et al. as a new metal-free route to silicone coatings and foams [19,20,21]. Brook et al. presented the first detailed studies of the P-R reaction between SiH-terminated polydimethylsiloxanes (H-PDMS-H) and tetraalkoxysilanes as cross-linkers [58]. The physical properties of the final silicone elastomer can be easily controlled with the selection of the starting polysiloxane molecular weight, the ratio of SiH to SiOR as well as the composition of the tetraalkoxysilane/trialkoxysilane cross-linker blend. Cure speed can be reduced by using alkoxysilanes with larger substituents, the addition of a solvent and the reduction of the catalyst concentration. The P-R reaction produces a volatile gas such as methane or ethane as a byproduct that can serve as a blowing agent and produce silicone foam during the curing process. Grande et al. used the P-R reaction to produce silicone foams from H-PDMS-H and tetraalkoxysilanes [59]. The properties of the resulting foam can be controlled by the molecular weight of the starting polymer, and the type and concentration of the cross-linking agent. The addition of a volatile solvent, which is hexane, allows for obtaining foam with a lower density.

Recently, Hickman et al. published a thorough study of the effect of the structure of the tetraalkoxysilane cross-linking agent on the properties of silicone elastomers obtained under P-R conditions [60]. The structure of these cross-linkers strongly influenced the morphology and properties of the cured elastomer. Electronic and steric effects have a great impact on the rate of the curing reaction. The use of tetra s-butoxysilane as the cross-linker was beneficial, leading to a fast network formation and good mechanical properties of the formed elastomer.

**Scheme 5:**
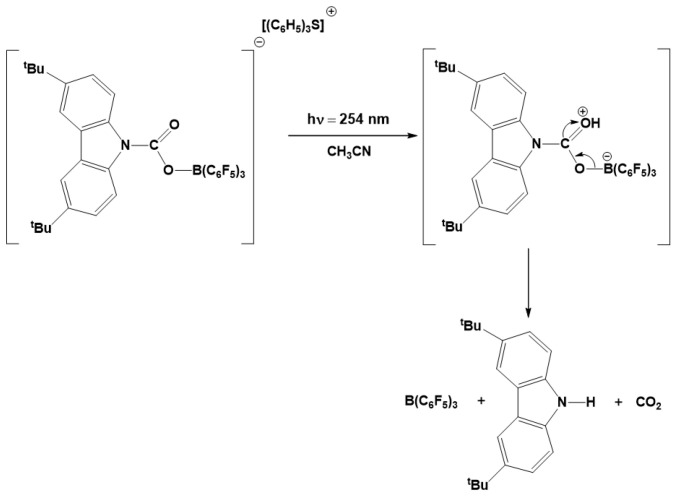
Decomposition under UV irradiation of carbamate borate triphenylsulfonium salt with the release of TPFPB.

The development of a catalytic system releasing the TPFPB molecule under exposure to UV radiation would allow for the formulation of silicone materials curing on demand using P-R reactions. Such a system based on the triphenylsulfonium salt of a carbomato borate was described and the initiation of the model P-R reaction using irradiation with UV light was demonstrated (Scheme 5) [61]. The later developed triphenylsulfonium and diphenyliodonium salts of hydridoborates ([HB(C_6_F_5_)_3_]^−^ [Ph_3_S]^+^, [HB(C_6_F_5_)_3_]^−^ [Ph_2_I]^+^) are simpler to prepare and even more efficient photo-generators of TPFPB [62].

### 3.2. Synthesis of Functionalized Polysiloxanes

**Scheme 6:**
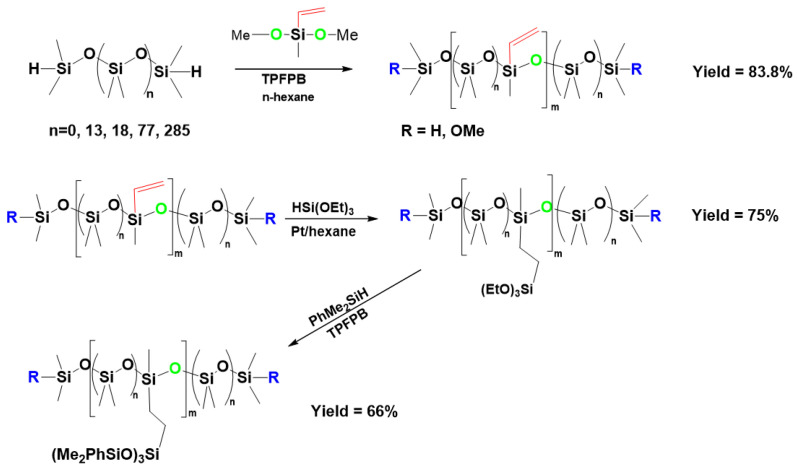
Example of a synthetic route to a dendritic polymer by combining the P-R reaction with Pt(0) hydrosilylation.

The P-R reaction is often used for the modification of polymers and larger molecules by introducing various organosilyl moieties to them. The P-R reaction is selective and more efficient than other silylation processes. The P-R reaction provides a high level of structural control that is needed for the synthesis of various siloxane copolymers with regularly distributed functional groups or blocks. Linear poly(dimethylsiloxane-co-vinylmethyldisiloxane) with the controlled spacing of vinyl functional groups was obtained in the P-R reaction of dimethoxymethylvinylsilane with linear polydimethylsiloxanes terminated with Si-H groups (Scheme 6) [63]. The hydrosilylation of this polymer with triethoxysilane followed by the P-R reaction with phenyldimethylsilane produced a highly branched polymer. The presented process shows a synthetic route to a dendritic polymer by combining the P-R reaction with hydrosilylation.

**Scheme 7:**
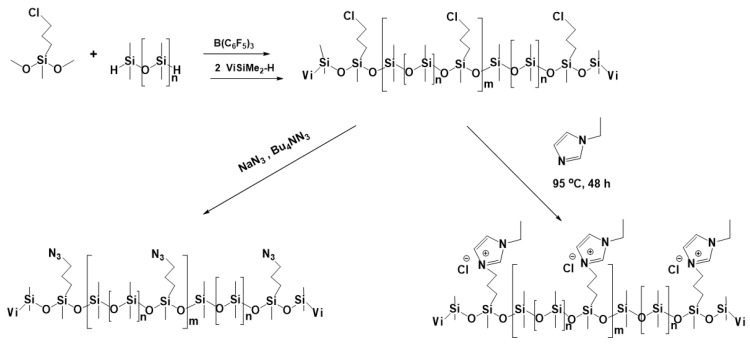
Synthesis of functional siloxane copolymers with spatially regularly spaced functional groups.

Polydimethylsiloxanes with spatially regularly spaced 3-chloropropyl side groups and allyl or vinyl end groups were prepared using the P-R polycondensation of 3-chloropropylmethyldiethoxysilane with oligodimethylsiloxane terminated by SiH groups. These copolymers, having SiOR end groups, were reacted with vinyl- or allyldimethylsilane to produce telechelic-functionalized polysiloxanes (Scheme 7) [64]. The subsequent functionalization of the pendant 3-chloropropyl groups was demonstrated to produce siloxane copolymers with azido and imidazolium functional groups placed regularly in the polymer chain.

**Scheme 8:**
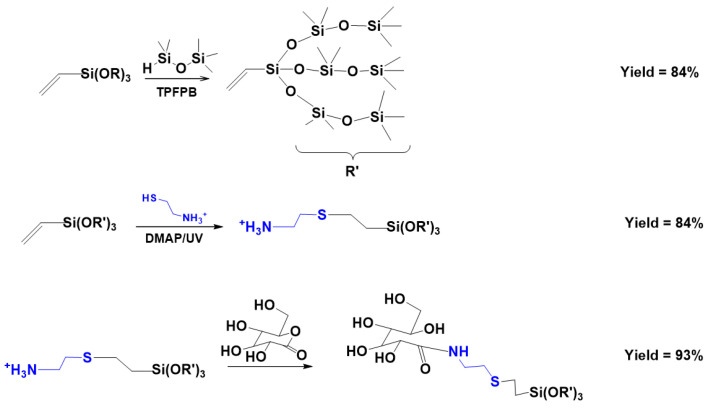
Synthesis of branched siloxane oligomers with aminoalkyl functional groups for modification of sugar lactones.

Vinyl-functionalized oligosiloxanes were prepared with the P-R reaction between pentamethyldisiloxane or 1,1,1,3,5,5,5-heptamethyltrisiloxane and vinyltriethoxysilane to produce corresponding tris(trioligosiloxy)vinyl silanes [65]. These oligosiloxanes were further functionalized with cysteamine to form aminoalkyl branched siloxanes. The presence of an aminoalkyl group allowed for their subsequent modification with various sugar lactones to obtain silicone surfactants of a natural origin (Scheme 8).

**Scheme 9:**
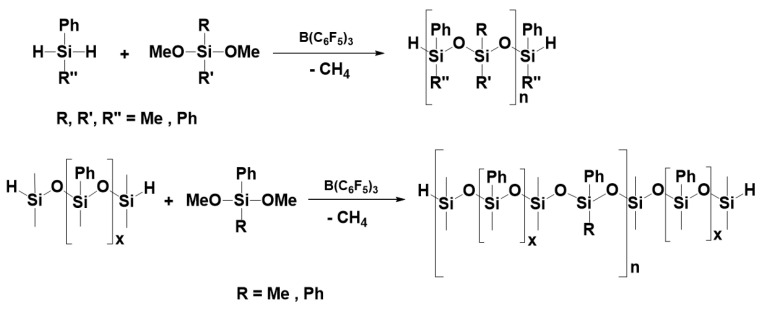
Synthesis of siloxane copolymers with regularly spaced phenylmethyl and diphenyl siloxane units.

Dimethylsiloxane copolymers with regularly spaced phenylmethyl and diphenyl siloxane segments were also prepared using the P-R reaction [66,67]. This process allows for obtaining copolymers with a high phenyl content including block and alternative copolymers (Scheme 9). Methods for the controlled synthesis of polysiloxanes that contain phenyl groups are needed due to a high thermal resistance, chemical stability and good mechanical properties at low temperatures of such polymers. These features are particularly important in manufacturing polysiloxane elastomers.

Muzafarov and co-workers introduced many phenyl groups to hyperbranched polyethoxyphenylsiloxanes using their reaction with dimethylphenylsilane catalyzed by TPFPB [68]. The obtained materials showed a high heat resistance, ranging from 405 °C to 480 °C, and interesting rheological properties.

**Scheme 10:**
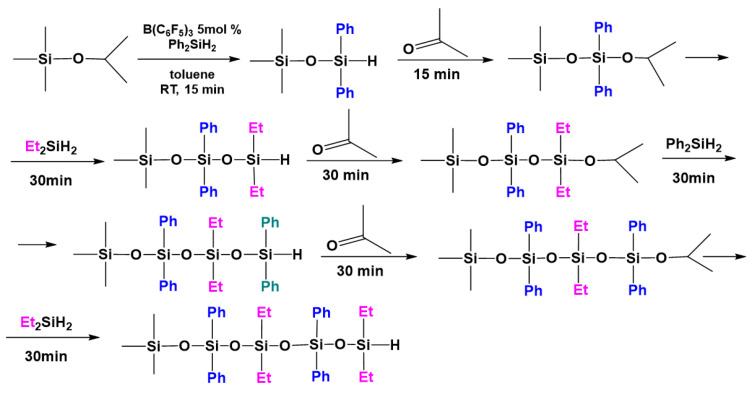
Synthesis of well-defined siloxane oligomers with a sequence-specific structure using a one-pot process involving two reactions catalyzed by TPFPB.

Sato et al. reported a creative approach to the synthesis of well-defined siloxane oligomers with a sequence-specific structure. They developed a simple one-pot process by combining two reactions catalyzed by TPFPB: the hydrosilylation of the carbonyl group of a simple ketone or aldehyde using a dihydrosilane (such as Et_2_SiH_2_) with the P-R condensation of the formed oligosiloxane with a terminal isopropoxy group with another dihydrosilane molecule (such as Ph_2_SiH_2_) [69]. A typical example of such a sequential methodology is shown in Scheme 10, where, starting from isopropoxytrimethylsilane, a linear pentasiloxane with alternating Ph_2_SiO and Et_2_SiO units was prepared in seven steps over 3 h with a final yield of 70%. The synthesis of well-defined branched and cyclic oligosiloxanes was also demonstrated using the same one-pot procedure. The authors anticipate the use of this methodology to obtain silicone materials with precisely tuned physical properties. The same group reported another one-pot synthesis of well-defined trisiloxanes by combining the iridium-complex-catalyzed hydrosilylation of an organosilyl acetate using dihydrosilanes to disilyl acetals with the TPFPB-catalyzed rearrangement of this compound to an ethoxy-functional disiloxane followed by the PR condensation of the formed disiloxane with another dihydrosilane molecule [70].

### 3.3. Synthesis of Polysiloxanes Functionalized with Thermolabile Groups and Novel Thermoset Materials

**Scheme 11:**
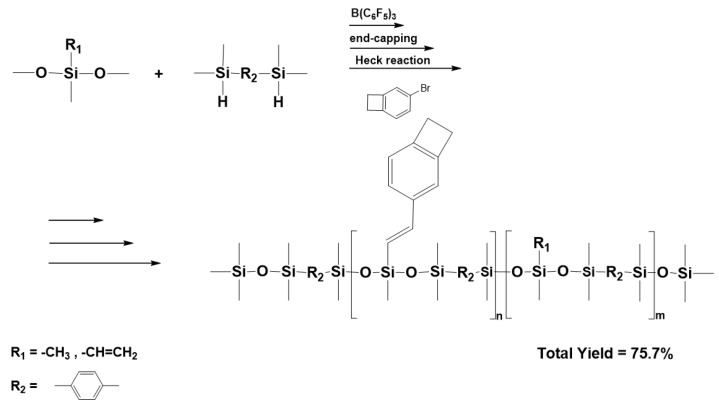
Synthesis of the polysiloxanes with pendant benzocyclobutene groups using the combination of the P-R reaction and Heck reaction.

The P-R reaction was used to obtain polysiloxanes functionalized with thermolabile groups, making it possible to obtain thermosetting resins that can be cured at elevated temperatures. The synthesis of a linear siloxane prepolymer of vinyl-functionalized curable resins was carried out with the P-R condensation method followed by the addition of thermo-curable benzocyclobutene groups (BCB) with the Heck reaction (Scheme 11) [71].

**Scheme 12:**
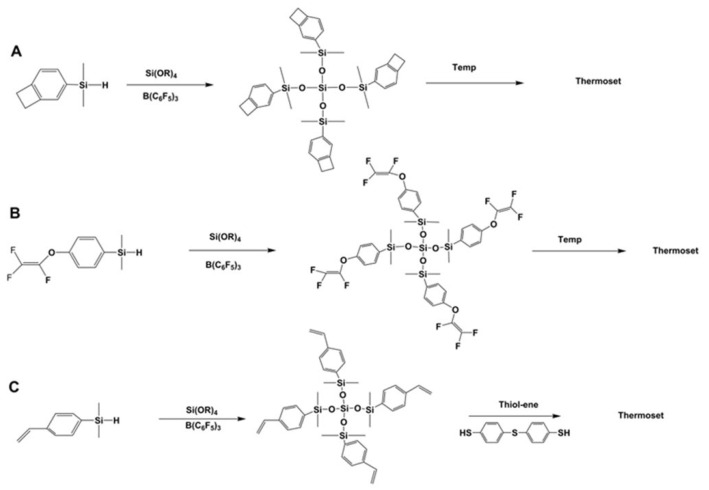
Three examples of the synthesis of multi-arm siloxanes with different thermolabile groups and their conversion to highly cross-linked carbo-siloxane materials. (**A**)—Benzocyclobutene group; (**B**)—Trifluorovinyloxyphenyl group; (**C**)—p-Vinylbenzene group.

Other recently developed strategies have also used the synthesis of siloxane macromers containing polymerizable functional groups. Multiarmed siloxane macromers with three or four benzocyclobutene functions were synthesized with the P-R condensation method and subjected to the thermal addition polymerization of the benzocyclobutene group to produce a highly cross-linked carbo-siloxane thermoset having a very good thermal stability and good dielectric properties (Scheme 12A) [72].

A siloxane star oligomer that is functionalized with trifluorovinyl ether groups capable of cyclodimerization at a high temperature was synthesized with a high yield using the P-R reaction of tetraethoxysilane with (trifluorovinyloxyphenyl)dimethyl silane, as shown in Scheme 12B [73]. The obtained compound was transformed with high-temperature treatment into a transparent, flexible siloxane film with a low dielectric constant and high UV absorption below 350 nm, which makes it a promising material for UV-blocking coatings and a matrix resin for microelectronics.

The same concept was applied to the preparation of the tetravinyl-functionalized siloxane star oligomer from tetraethoxysilane and p-vinyl(hydrodimethylsilyl)benzene (Scheme 12C). The resulting macromer was converted to a high-refractive-index siloxane sulfide aromatic cross-linked material of a high thermal stability using thiol-ene addition with 4,4′-thiodibenzenethiol [74].

**Scheme 13:**
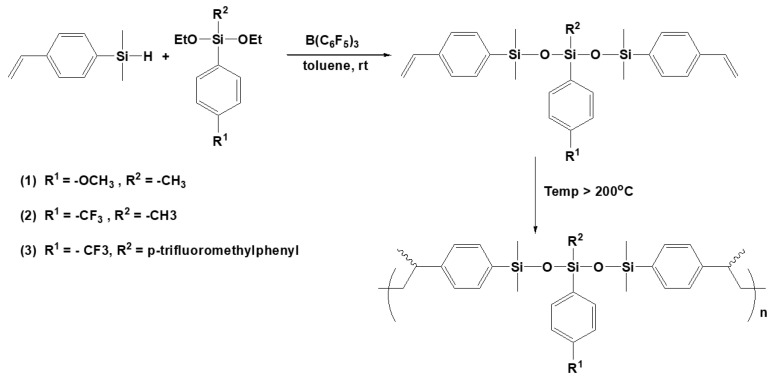
Synthesis of siloxane macromers with trifluoromethylphenyl group and two styryl functions; their conversion to highly cross-linked material with excellent thermal stability and good mechanical and dielectric properties.

Siloxane macromers containing a trifluorophenyl group and substituted by two styryl functions were successfully synthesized with the P-R reaction. The thermal free-radical polymerization of these styryl groups led to densely cross-linked hydrophobic material with a high thermal stability and good dielectric properties (Scheme 13) [75]. The same authors synthesized, with a similar method, the siloxane macromer substituted at silicon by a bis-3,5(trifluoromethyl)phenyl group, which had two benzocyclobutene and one vinyl thermo-reactive functions [76]. The thermal cycloaddition of this compound produced a cross-linked hydrophobic material with an excellent thermal stability and good mechanical and dielectric properties.

### 3.4. Synthesis of Branched and Hyperbranched Polysiloxanes and MDTQ Resins

**Scheme 14:**
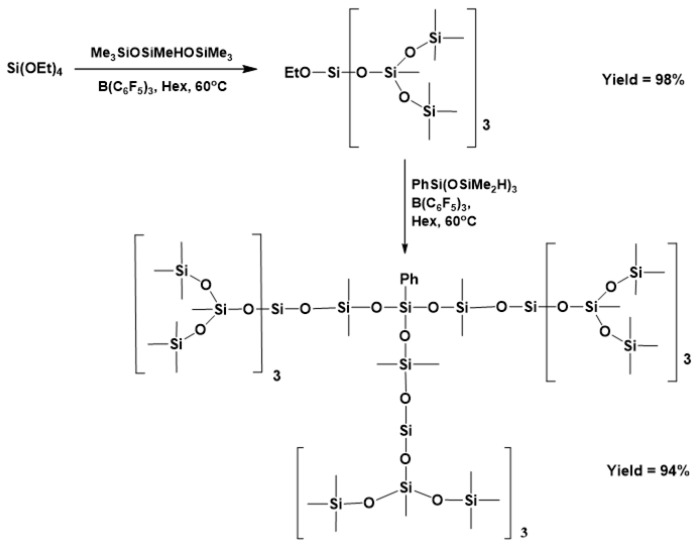
Synthesis of the regular branched polysiloxanes of a controlled structure with the two-step P-R process.

The P-R reaction is often a unique method for the synthesis of branched polysiloxanes with a high degree of control of the macromolecule microstructure. Brook et al. showed that branched polsiloxanes of a complex 3D architecture may be built with a high yield [26,77]. Star oligosiloxane tetrakis(pentamethyldisiloxane)silane was obtained with the P-R condensation of pentametyldisiloxane with tetrapropoxysilane with a 97% yield under mild conditions, and the oligomer with an even more branched structure was obtained at 60 °C with the yield of 94% (Scheme 14). Under the conditions used in these reactions, no products of the metathetic exchange of functional groups were detected [77].

**Scheme 15:**
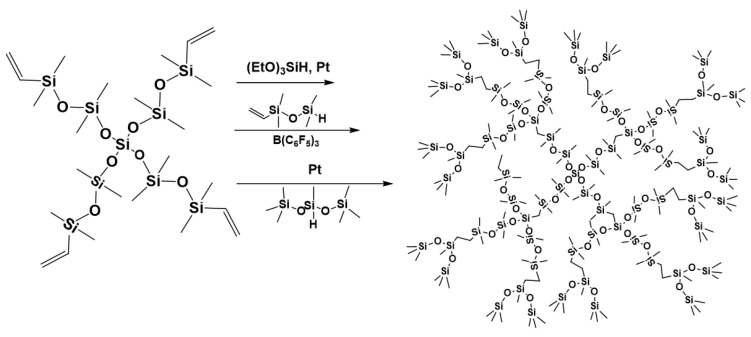
Example of the synthesis of dendrimers by combining the P-R reaction with Pt(0)-catalyzed hydrosilylation.

The same research group demonstrated that P-R condensation and platinum-catalyzed hydrosilylation are orthogonal reactions, which enable the synthesis of silicone dendrons and dendrimers of a high molecular weight with an excellent precision and high yield (Scheme 15) [78]. The P-R reaction was also used to synthesize a large number of vinyl-terminated dendrons, which were then grafted onto linear poly(dimethyl-co-hydromethyl)siloxanes (PDMHMS) with platinum-catalyzed hydrosilylation [79]. Only about 60% of the available SiH groups were grafted by these branched compounds, which was explained by the presence of clusters of Si-H units in the polymer chain and the resulting steric reasons.

**Scheme 16:**
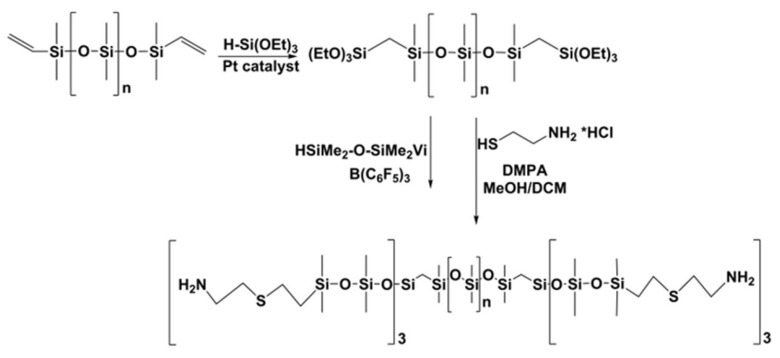
Example of the synthesis of the dendritic polysiloxane with chelating multidentate ligands.

The combinatorial (in tandem) use of the P-R reaction and tiol-ene addition led to the preparation of dendritic polysiloxane with chelating multidentate ligands able to capture a variety of metal ions (Scheme 16). These dendrons were used as coatings, protecting the surfaces of silicone elastomers against pathogens [80].

**Scheme 17:**
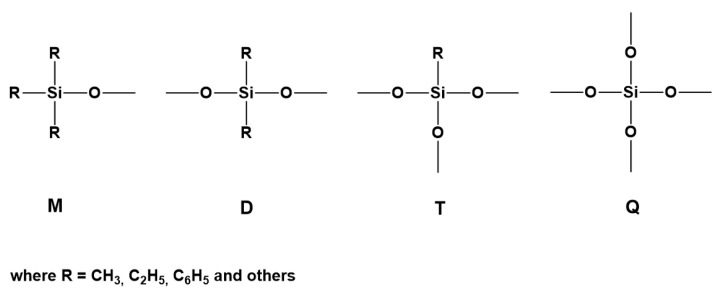
Symbols used to describe the functionalities of the structural units in siloxane materials.

Polysiloxanes containing SiO_4/2_ (Q) and RSiO_3/2_ (T) branched segments, R_2_SiO_2/2_ (D) bridges and R_3_SiO_1/2_ (M) end groups are commonly referred to as MDTQ resins (Scheme 17) and are widely used in silicone materials to modify their properties [8,81,82].

**Scheme 18:**
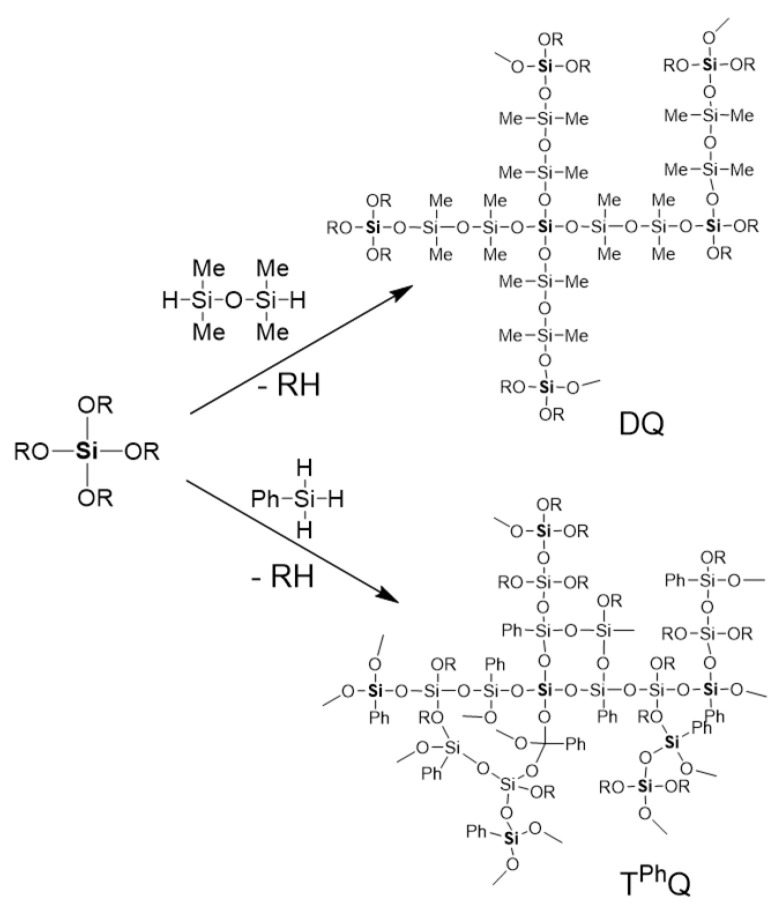
Synthesis of highly branched alkoxy-functional DQ and T^Ph^Q resins.

The P-R reaction is a useful method of the synthesis of highly branched reactive polysiloxanes using three or tetrafunctional monomers (Scheme 18). The polymers obtained were able to undergo further reactions. The P-R polycondensation of phenylsilane with tetraethoxysilane produced hydrophobic soluble T^Ph^Q resin containing phenyl and reactive alkoxy groups. The resin showed long-term stability because it did not contain silanol groups [83]. The P-R polycondensation of tetraethoxysilane with 1,1,3,3-tetramethyldisi loxane yielded a hyperbranched stable polyalkoxysiloxane of a high molecular weight free of silanol groups (Scheme 18) [84].

Highly reactive poly(phenyl-substituted siloxanes/silsesquioxanes) were also obtained from short linear polyhydromethylsiloxane and diphenyldimethoxysilane or diphenyldiethoxysilane with the P-R process [85]. The P-R reaction proceeded together with the exchange of reactive SiH and SiOR groups, leading to bridges composed of phenyl-substituted disiloxane units. The final polymers had a high refractive index and were rich in phenyl and Si-H and alkoxy reactive groups. They could serve as a cross-linking agent for addition- or condensation-cure packaging materials for light-emitting diodes.

**Scheme 19:**
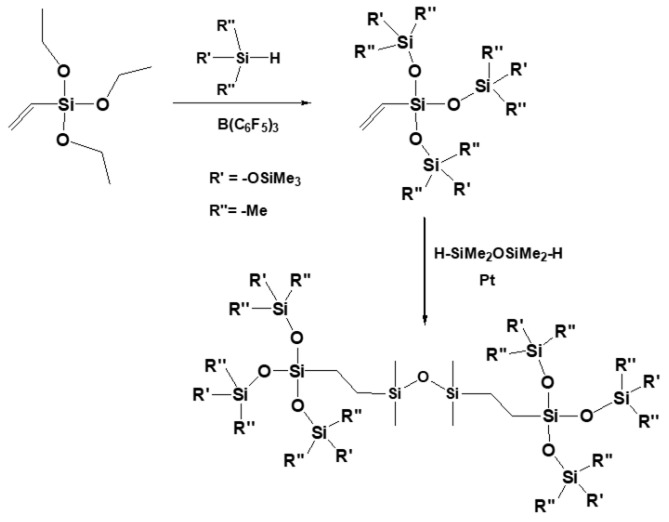
Example of the preparation of MTD resin with the combination of the P-R reaction and hydrosilylation process.

A library of hyperbranched dendritic polysiloxanes of various MDT and MDQ structures were prepared with the combination of the P-R and hydrosilylation methods (Scheme 19). These resins can be used as additives in silicone gels to control their properties such as adhesion and transparency [86].

**Scheme 20:**
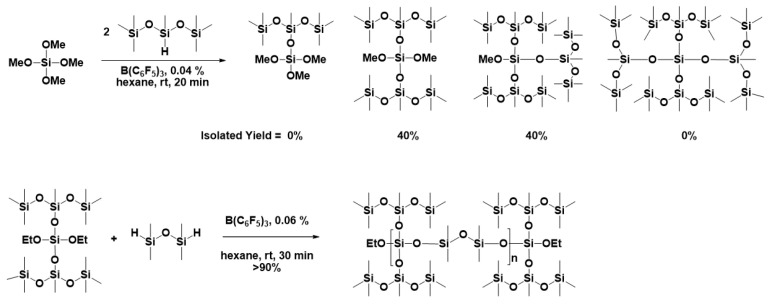
Example of the preparation of the MDTQ resins with the two-step P-R reaction.

Liquid MDTQ resins containing alternating Q and T segments with D bridges were obtained in a two-step synthesis using the P-R reaction [87]. First, well-defined branched siloxane oligomers with M, T and Q units that had the remaining two alkoxy functions were prepared and isolated with distillation, then chain extension was performed using P-R polycondensation with linear SiH-terminated oligomers, such as 1,1,3.3-tetramethyldisiloxane (Scheme 20). Alternatively, the P-R reaction of the same oligomers having two alkoxy functions with 1,4-bis(dimethylsilyl)benzene produced alternating QT-silphenylene polymers. The obtained polymers had short branches and, consequently, a relatively low viscosity. Their use as additives to silicone elastomers was proposed.

**Scheme 21:**
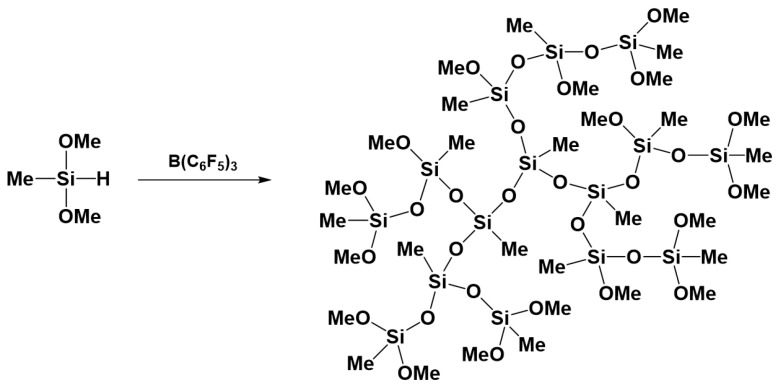
Example of the one-pot synthesis of the hyperbranched polysiloxanes from dimethoxy metylsilane.

Hyperbranched polysiloxanes are readily obtained with the P-R polycondensation of polyfunctional monomers of the AB_2_ type, where A and B are SiH and SiOR groups, respectively [19,20,21]. The slow addition of dimethoxymethylsilane to toluene, containing catalytic amounts of TPFPB, was found to lead to a rapid reaction with gas evolution. The ^1^H NMR of the final viscous fluid showed a complete SiH conversion and 50 mol% conversion of the Si-OMe groups. The ^29^Si NMR showed the formation of a hyperbranched polymer with T units and with Si-OMe end groups (Scheme 21). A small amount of CH_3_Si(OCH_3_)_3_, which is a functional group exchange product, was also detected. These highly functional polysiloxanes can be used as a cross-linking agent or an adhesion promoter additive.

A similar synthetic procedure was recently used by Shi et al. for the preparation of relatively low molecular weight branched alkoxy-functional polysiloxane. Subsequently, the obtained polymer was reacted with benzocyclobuten-4-yl-dimethylsilane with a second P-R reaction [88]. The obtained polymer was cured at a temperature gradually elevated to 290 °C, at which cyclobutene groups underwent (4 + 2) cycloaddition to form a six-member ring unit. The obtained material with very good dielectric properties and excellent behavior at high temperatures shows potential as an innovative encapsulating material for advanced electronic applications.

Fast-curing silicone resins were prepared with the P-R reaction of tetrakis(dimethylsiloxy)silane with 1,3-dimethyltetraethoxydisiloxane [89]. The obtained resins were used as a hydrophobing agent, which can be conveniently applied to Whatman paper using ink-jet printing. The developed technology can be used as an inexpensive process for the rapid prototyping of various assays.

### 3.5. Preparation of Polysiloxanes with a Regular Complex Structure with Cyclic or Cage Groups in the Backbone

**Scheme 22:**
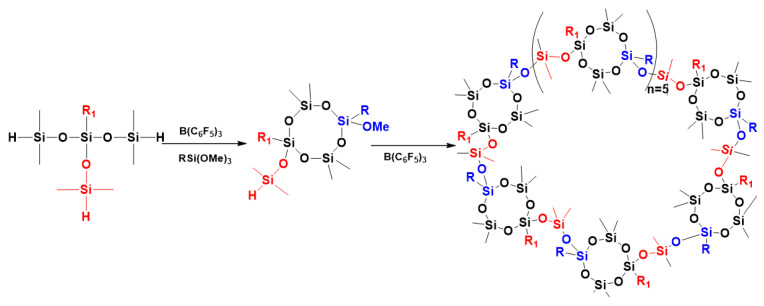
Example of the synthesis of cyclic polycyclotetrasiloxane polymers with the P-R process.

The P-R reaction enables the synthesis of polysiloxanes containing regular cyclic or cage units and using them for the construction of complex architectures with various regular structures of polymer backbones. Macrocyclic siloxanes having chains formed with siloxycyclotetrasiloxane units were successfully constructed in a one-step process by selecting the appropriate SiH and SiOR reagent, solvent and reaction conditions (Scheme 22) [90].

**Scheme 23:**
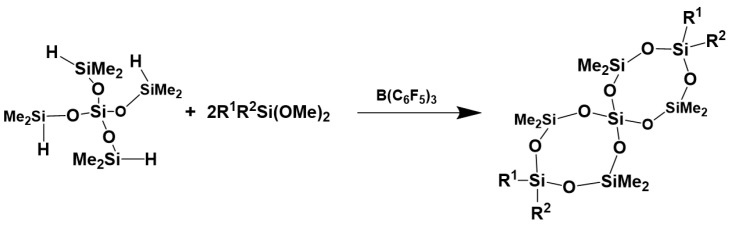
Example of the synthesis of spirocyclosiloxanes in cyclohexane in the presence of TPFPB.

A similar approach was used to prepare spirocyclosiloxanes that were obtained from commercially available tetrakis(dimethylsilyloxy)silane and two molar equivalents of various diorganodimethoxysilanes in the P-R reaction (Scheme 23). Interestingly, the choice of solvent plays a key role here. The desired spirocyclosiloxanes were obtained in high yields when cyclohexane was used as the solvent. The same reaction performed in methylene chloride did not produce a significant amount of spirocyclosiloxanes [91].

The anionic copolymerization of such spirocyclosiloxanes with various cyclic siloxanes leads to the formation of highly transparent cross-linked elastomers [91].

**Scheme 24:**
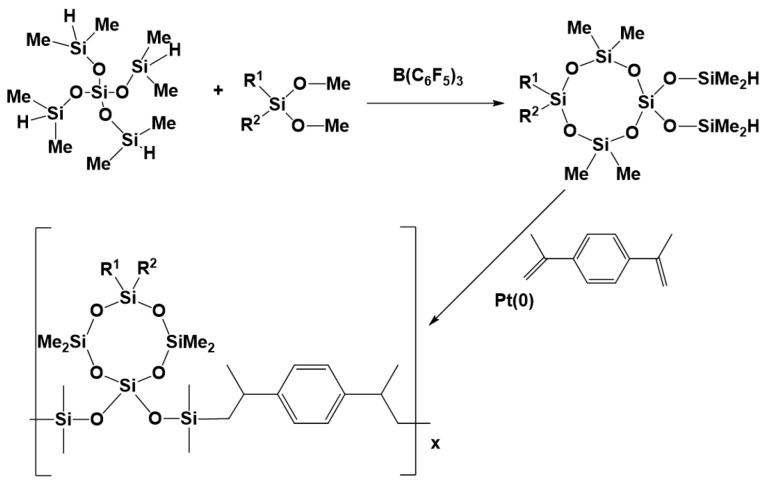
Synthesis of cyclotetrasiloxanes with two side SiH groups and their conversion to a linear polymer via Pt-catalyzed hydrosilylation process with preserved cyclotetrasiloxane structure.

An analogous P-R reaction of tetrakis(dimethylsilyloxy)silane with one molar equivalent of various diorganodimethoxysilanes in methylene chloride produces cyclotetrasiloxane with two pendant SiH groups (Scheme 24) [92]. The subsequent P-R reaction of this new monomer with methoxy- or silanol-stopped polydimethylsiloxanes produces linear polydimethylsiloxanes with incorporated cyclotetrasiloxane rings. Various polymers containing a cyclotetrasiloxane ring in the main chain can also be prepared by the hydrosilylation of the same monomer with difunctional olefins (Scheme 24).

Macrocyclic siloxanes composed of spirosiloxane units linked by compounds containing a reactive SiH group were synthesized with the P-R polycondensation of a spirosiloxane that had two isopropoxysilane substituents with phenylsilane, PhSiH_3_ [93]. Remaining SiH groups were used for the introduction of various functions to the polymer by hydrosilylation.

**Scheme 25:**
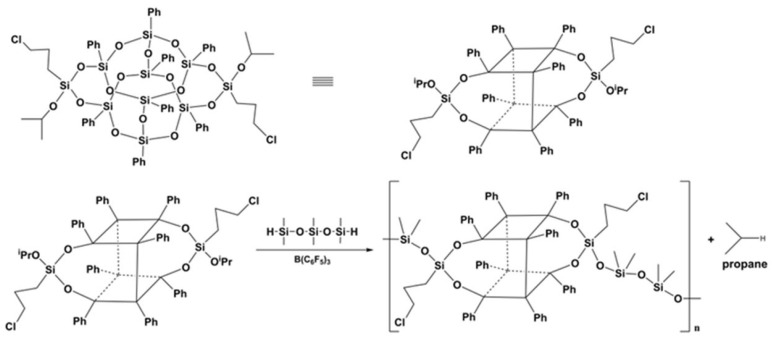
Synthesis of the poly(siloxane/double-decker silsesquioxane) copolymer.

Di[(3-chloropropyl)isopropoxysilyl]-bridged double-decker octaphenylsilsesquioxane was reacted with 1,1,3,3,5,5-hexamethyltrisiloxane in the presence of TPFPB in toluene at ambient conditions to produce a poly(siloxane/double-decker silsesquioxane) copolymer in a high yield (Scheme 25) [94]. The obtained copolymer with 3-chloropropyl side groups was subsequently converted to bromine-containing poly(siloxane/double-decker octaphenylsilsesquioxane) via halogen exchange with only a small degradation of the siloxane chain.

**Scheme 26:**
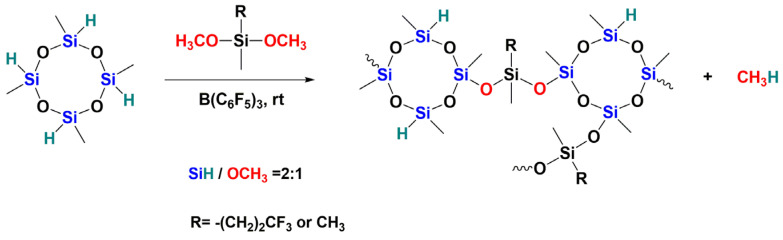
Synthesis of liquid polysiloxane with preserved cyclotetrasiloxane rings and reactive Si-H groups.

The effectiveness of the P-R reaction in the construction of assemblies composed of larger siloxane molecules was demonstrated by Mitsuishi et al., who carried out the P-R polycondensation of 1,3,5,7-tetramethylcyclotetrasiloxane, (D^H^_4_), with dimethyldimethoxysilane or 3-(trifluoropropyl)methyldimethoxysilane [95]. The reaction produced a liquid polymer containing cyclic siloxanes with SiH groups bridged by disiloxane units (Scheme 26). The authors were able to suppress the competitive hydride-transfer ring-opening polymerization of the D^H^_4_, which could lead to premature gel formation, by selecting relatively low reagent concentrations and limiting the reaction time to 2 h. The liquid polymer with reactive Si-H groups was then converted into a free-standing transparent film with thermal curing in air. The resulting transparent film had an excellent thermal stability up to temperatures above 630 °C and elastic moduli greater than 74 MPa. The dielectric properties of the film can be adjusted by selecting the substituents of the starting organodimethoxysilane. The developed polymer can be used to produce various functional materials.

Yoshikawa and co-workers reacted two types of cyclododecasiloxanes, one substituted by SiOR groups and the other with SiH groups, under P-R conditions, forming a spatial network in which the cyclic structure of cyclododecylosiloxanes was preserved [96].

**Scheme 27:**
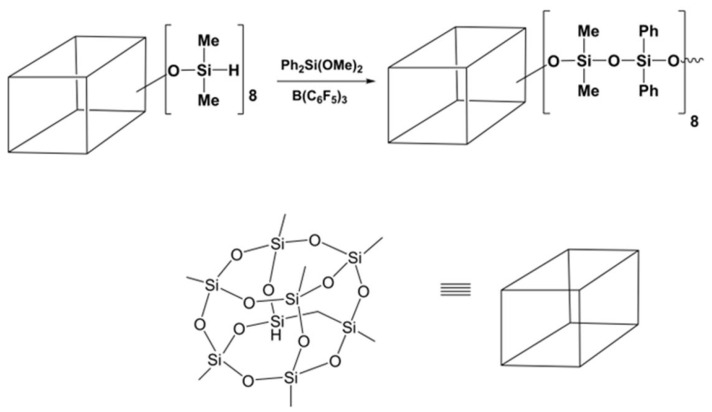
Synthesis of the super large hyperbranched siloxane structures from Q_8_M^H^_8_ silicate.

Cage cubic Q_8_ silicate substituted by OSiMe_2_H (M^H^) groups in all its eight corners (Q_8_M^H^_8_) underwent the P-R polycondensation with diphenyldimethoxysilane, as shown in Scheme 27 [97]. The result was a hyperbranched polymer with a very high molecular weight, Mw = 1–10 M, containing over 2000 Q_8_ cage units. The authors claimed to have obtained huge individual macromolecules as large as 10 nm.

Laine and co-workers carried out a similar P-R reaction of octahedral Q_8_M^H^_8_ silicate with tetraethoxysilane and some organosilicon compounds with a triethoxysilyl moiety. These reactions resulted in 3D networks that formed microporous materials with large pore surfaces [98].

### 3.6. Modification of Nature-Based Materials Using the P-R Reaction

The P-R reaction was successfully used for the modification of natural materials [99,100,101]. Trimetoxysilyl groups were introduced to soybean oil via an ene reaction. The silylated soybean oil was then mixed with a blend of two low-viscosity siloxane oils, SiH-terminated polydimethylsiloxane (DMS-H03) with poly(hydromethylsiloxane) (HMS-992), using a speedmixer and subjected to the P-R reaction. Various cross-linked foams and elastomers that contained up to 76% soybean oil were produced. The resulting materials did not match the mechanical properties of polyurethane foams, but they showed a better resistance to combustion and, if ignited, burned cleanly, with little smoke and no dripping in comparison to polyurethane foams [99].

The P-R reaction was used to modify lignin particles. Small particles of softwood lignin (<70 μm) were mixed with SiH-terminated short-chain polydimethylsiloxane, tetraethoxysilane and a catalytic amount of TPFPB. The P-R reaction proceeded between tetraethoxysilane and polydimethylsiloxane, forming a network and with functional groups on the surface of the lignin particles, producing a silicone elastomer reinforced with the lignin particles [100].

**Scheme 28:**
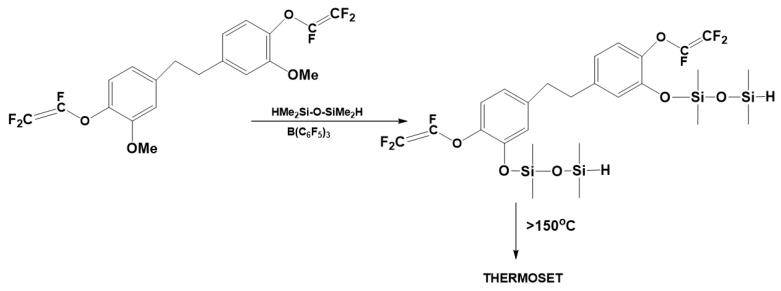
Synthesis of the fluoro-containing polysiloxane thermoset resin from vanillin.

Lignin-derived vanillin was dimerized and transformed to bis(trifluorovinyl)ether. The methoxy groups of this compound were used in a P-R polycondensation with 1,3-dihydrotetramethyldisiloxane, producing fluoro- and siloxane-containing resin that could undergo further thermal cross-linking with the cyclodimerization of trifluorovinyloxy groups, as shown in Scheme 28 [101]. The obtained bio-derived siloxane film showed good transparency, mechanical strength and excellent acid stability.

### 3.7. Synthesis of Polysiloxanes with Organic Fragments in Main Chains

**Scheme 29:**
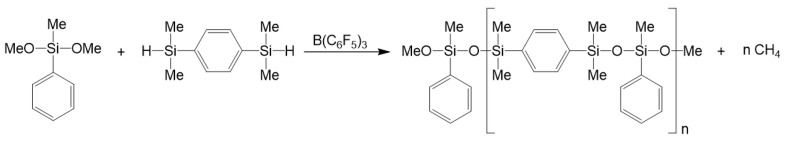
Synthesis of the siloxane copolymer with 1,4-bis(dimethylsilyl)benzene units.

The P-R reaction was used to synthesize copolymers that had both siloxane and organic fragments in the main polymer chain. Rubinsztajn and Cella prepared a series of polycondensations of diorganodimethoxy silanes and bis(dimethylmethoxysilyl)benzene with 1,4-bis(dimethylsilyl)benzene and 4,4′-bis(dimethylsilyl)diphenylether (Scheme 29). They obtained linear siloxane copolymers having benzene and dibenzo ether moieties in the polymer chains, with an average molecular weight ranging from 10^3^ to 5 × 10^4^ g/mol [22].

**Scheme 30:**
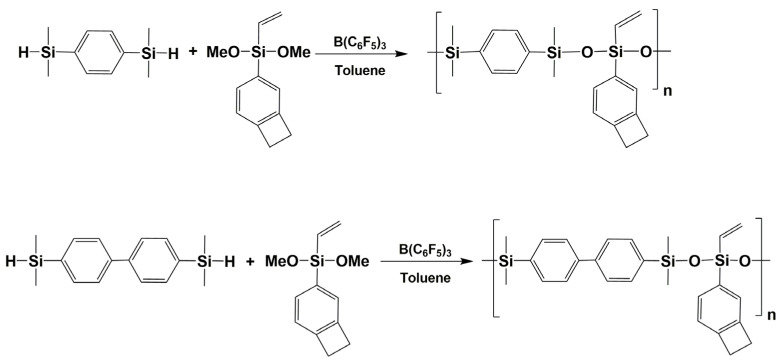
Synthesis of the silphenylenesiloxane copolymers with pendant vinyl and benzocyclobutene groups.

A similar reaction between vinylmethyldimethoxysilane and 1,4-bis(dimethylsilyl)- benzene was later performed to obtain a vinyl-substituted silphenylenesiloxane copolymer [102]. The P-R reaction also produced curable siloxane oligomers with a silphenylene or silbiphenylene unit and pendant vinyl and benzocyclobutene (BCB) functional groups (Scheme 30) [103]. These oligomers were cured above 200 °C to form a highly cross-linked thermoset of good dielectric and mechanical properties.

**Scheme 31:**
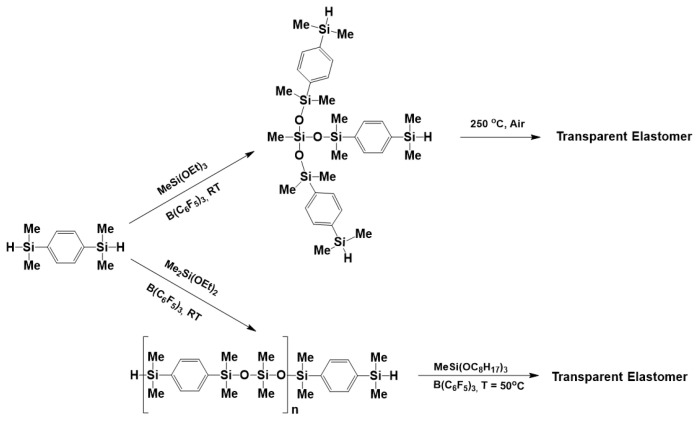
Two examples of the synthesis of transparent silphenylene elastomers developed by Brook and co-workers.

The synthesis of transparent silphenylene elastomers is challenging due to the polymer’s tendency to self-associate by stacking together aromatic rings. Brook and co-workers reported several methods of preparing transparent silphenylene elastomers [104]. These methods used branched macromers or introduced either siloxane units or catechol to the silphenylene chains. Trifunctional or tetrafunctional macromers were synthesized using the P-R condensation of tri- or tetramethoxysilanes with an excess of 1,4-bis(hydrodimethylsilyl)benzene. These Si-H functional macromers, after curing in air at 250 °C, produced transparent elastomers, as shown in Scheme 31. Another method involved mixing a hydride-terminated silphenylene–siloxane copolymer with trioctyloxyvinylsilane and a catalytic amount of TPFPB in a planetary centrifugal mixer. The resulting blend was cured to a transparent elastomer at 50 °C with the P-R reaction.

Hyperbranched polycarbosiloxanes were produced with the P-R condensation of SiH-terminated monomers: 1,4-bis(hydrodimethylsilyl)benzene, 4,4′-bis(hydrodimethyl silyl)-1,1′-biphenyl and 1,1′-bis(hydrodimethylsilyl)ferrocene with methyl- or phenyltriethoxysilane. The pyrolysis of the ferrocene-containing polymer at 900 °C under nitrogen produced a ceramic material with a good yield above 45%. The obtained ceramic material exhibited a good magnetizability [105].

**Scheme 32:**
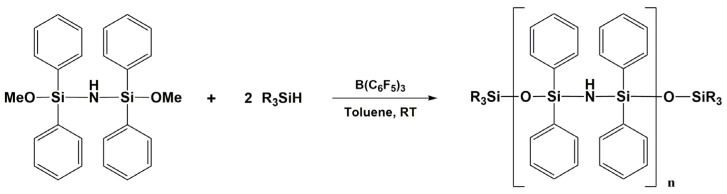
Conversion of 1,3-dimethoxytetraphenyldisilazane to polysiloxazanes with the P-R process.

The first example of the P-R reaction of compounds containing Si-N-Si units was reported by Xu and co-workers. They studied the P-R reaction of 1,3-dimethoxytetraphenyldisilazane with several triorganosilanes, R_3_SiH, where R = Et, Me, Vi and Ph in the presence of a relatively high concentration of TPFPB (0.5 to 3 mol%). They found that the expected dehydrocarbonative condensation was strongly accompanied by the functional group exchange process. As a result of this competition, several R_3_Si-terminated oligomeric products with different numbers of Si-N-Si-O repeating units were obtained. Subsequently, they prepared polysiloxazanes with molecular weights ranging from 3.5 to 44.7 [102] and a relatively narrow molecular weight distribution by reacting 1,3-dimethoxytetraphenyldisilazane with SiH-terminated oligodimethylsiloxanes in the presence of TPFPB. The obtained results showed that the weakly basic nitrogen of tetraphenyldisilazane and the steric effect of four phenyl groups prevent it from forming a strong complex with TPFPB. This allows the TPFPB to interact with the SiH groups and facilitates the P-R reaction (Scheme 32) [106].

It was shown that the P-R reaction can also be performed in the presence of less basic functional groups such as thiols and sulfide [107]. Tan and co-workers applied this knowledge to make various polyoxyethylene–polyoxypropylene copolymers with different siloxane end groups. They tested these materials as surfactants and found that the micellization process was strongly affected by the structure of the hydrophobic siloxane end groups [108]. The P-R reaction was also used to obtain a branched siloxane head group of the anionic-sulfonate surfactant (Et_3_SiO)_2_EtSi-CH_2_CH_2_SO_3_K [109].

## 4. Hydride Transfer from Si to C: P-R Reaction Leading to SiOC

**Scheme 33:**
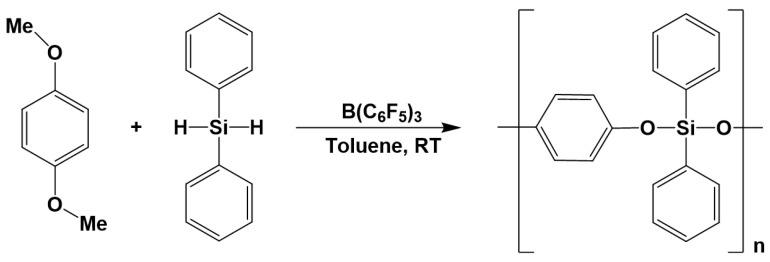
Synthesis of polyaryloxysiloxanes with the P-R condensation of hydroquinone dimethyl ether and diphenylsilane.

TPFPB is a catalyst that can make hydrosilanes react with both aromatic and aliphatic ethers. In this reaction, a hydrogen atom from silicon transfers to the carbon atom next to the oxygen atom in the ether group, breaking the ether bond and creating a new silaether bond between silicon and oxygen. This process is also called the P-R reaction, and it can be used to make polysilaethers and siloxane organic copolymers. Cella and Rubinsztajn were the first to report this reaction between hydroquinone dimethyl ether and diphenylsilane or 1,4-bis-dimethylsilylbenzene to produce corresponding polyaryloxysiloxanes, as shown in Scheme 33 [110].

**Scheme 34:**
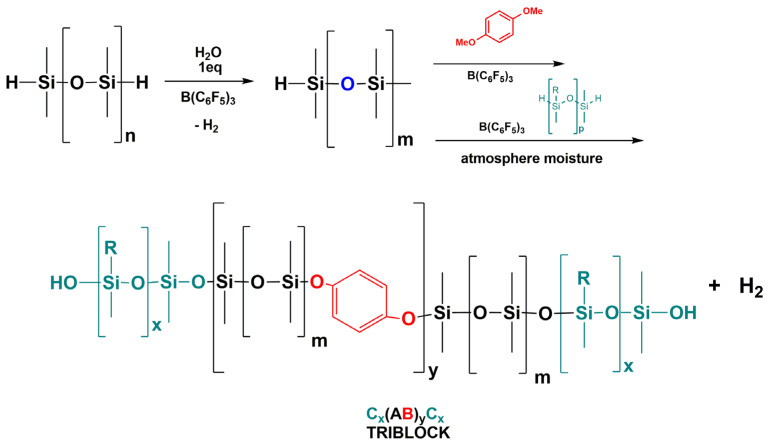
Synthesis of the tri-block polysiloxane with hydroquinone units (C_x_(AB)_y_C_x_ using the two-step P-R process.

Recently, Brook et al. extended this chemistry to make bi- and tri-block copolymers of polysiloxane with hydroquinone by reacting SH-terminated polysiloxanes with hydroquinone dimethyl ether (Scheme 34) [111].

**Scheme 35:**
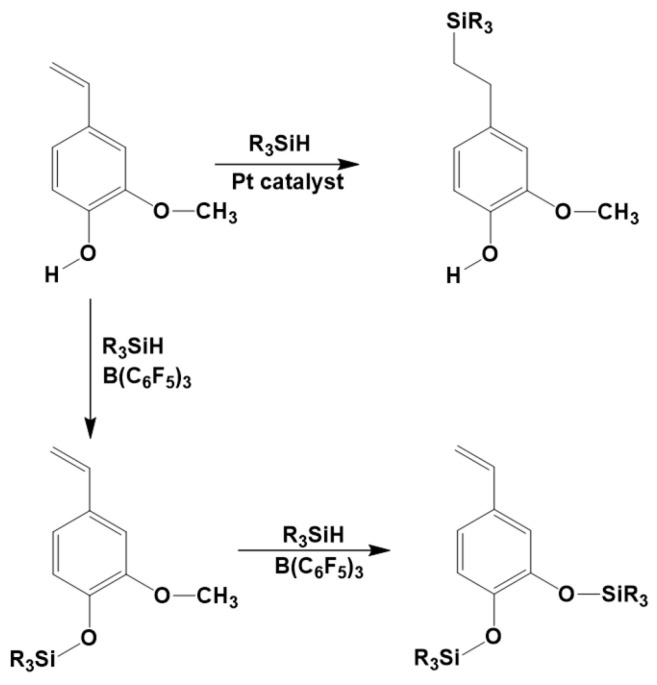
Reactivity of the eugenol molecule in the hydrosilylation process and P-R reaction.

Eugenol is a molecule that has a benzene ring with methoxy, hydroxyl and vinyl groups attached to it. The hydroxyl and methoxy groups can react with polysiloxanes that have SiH groups at their ends, using TPFPB as a catalyst. The hydroxyl group is more reactive than the methoxy group in the P-R reaction, so they can react separately (Scheme 35). The vinyl group can undergo a hydrosilylation reaction with polysiloxanes that have SiH groups at their ends, using a Pt(0) complex as a catalyst. By combining these three reactions, it is possible to make siloxane-based polymers that have different structures, such as linear, branched or networked [112]. These reactions were also used to produce silicone/eugenol resins that can be used to fabricate films that have a high refractive index. These resins can be used as LED encapsulants [113].

**Scheme 36:**
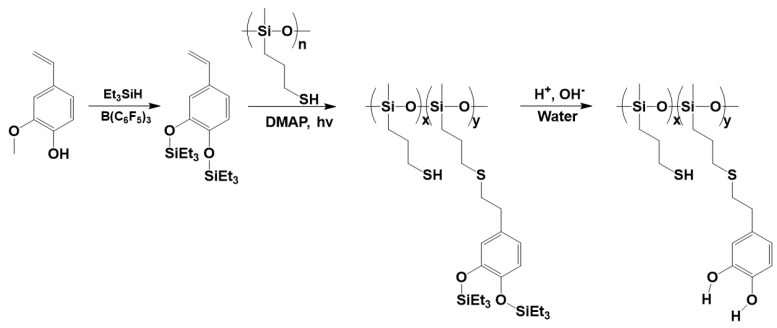
Synthesis of the siloxane copolymers with pendant catechol groups.

The Hawker group reported the functionalization of siloxane polymers with pendant catechol moieties from readily available eugenol via the thiol-ene reaction [114]. In order to improve the efficiency of this process, the protection of eugenol against oxidation during the thiol-ene reaction is required, which was conducted using the hydrosilylation of this compound with triethylsilane under P-R conditions (Scheme 36). The triethylsilyl protecting group can be removed under mild acidic or basic conditions. The resulting polysiloxanes with various degrees of catechol functionalization could serve as materials for the fabrication of 3D microstructures with a tailored adhesion to various oxide surfaces.

The same process has found an application in organic synthetic chemistry. Alkoxy groups as part of alkyl aryl ethers are one of the more common protecting groups of phenolic compounds. The P-R reaction is a mild and facile method for converting aryl-alkyl ethers to aryl-silaethers and subsequent deprotecting phenolic OH groups. It has an advantage over other deprotection methods. It liberates phenol at an ambient temperature with a high yield and tolerates many other functional groups [115,116].

**Scheme 37:**
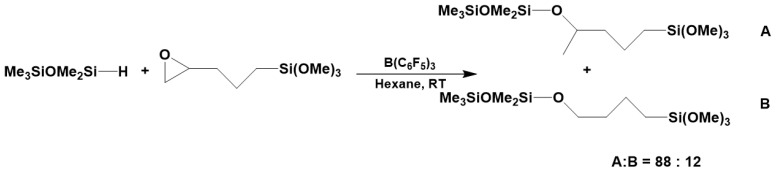
Opening of the epoxide ring using siloxanes with Si-H functional groups in the presence of TPFPB.

Brook et al. used the P-R reaction to open epoxides. They reported that the reaction of 3-(glycidoxy)propyltrimethoxysilane with pentamethyldisiloxane in the presence of a catalytic amount of TPFPB in hexane only produced products of a reductive epoxide ring opening. Two epoxide ring-opening products are possible, Scheme 37 A and B. They estimated that structure A is a dominated product [117].

Laine and their team followed up on this work and demonstrated that the reaction of various diepoxides (e.g., bisphenol A diglycidylether) with tetramethyldisiloxane produces a linear polysilaether. The analogous reaction of diepoxides with reagents containing multiple SiH groups, including Q_8_ cubic silicate and D^H^_4_ or D^H^_5_ cyclic monomers, created cross-linked materials with an ordered three-dimensional structure [118]. The chemistry presented could lead to a new amine-free curing system for epoxy resins.

**Scheme 38:**
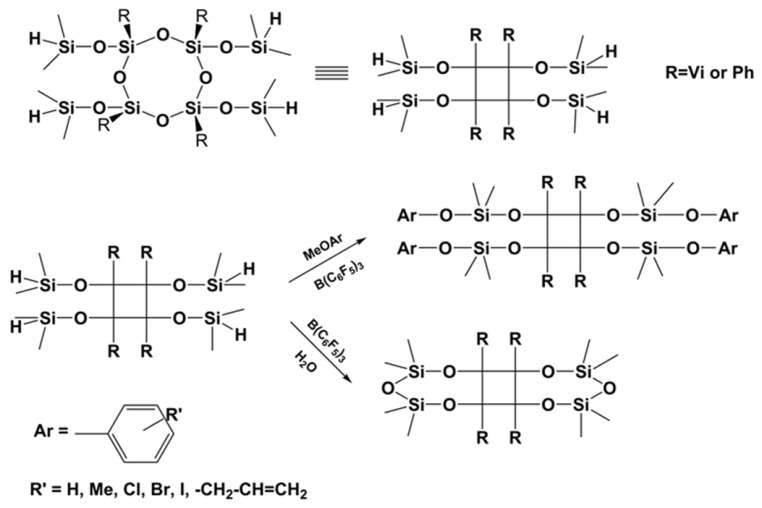
Synthesis of Janus-type silsesquioxanes with P-R reactions.

Unno and co-workers prepared all cis-tetrasiloxycyclotetrasiloxanes, calling them “Janus ring siloxanes”, with the general structure [RSi(OR′)O]_4_, where R = Vi or Ph and R′ = SiMe_2_H. They demonstrated that these compounds can be further functionalized with various aryl anisoles (MeOAr) via the P-R reaction (Scheme 38) [119,120]. The same Janus ring siloxanes undergo cyclization to the tricyclic ladder siloxane in the presence of water under P-R conditions. The newly prepared compounds are considered as precursors to well-defined Janus-type silsesquioxanes and functional porous materials.

**Scheme 39:**
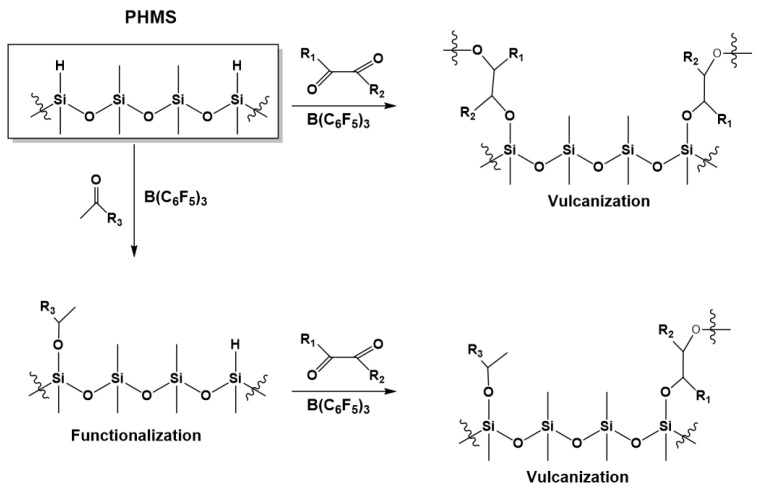
New process of the cross-linking of PDMHMS developed by the Hawker group.

Hawker and co-workers showed a new way to make cross-linked silicone elastomers by using TPFPB as a catalyst for the reaction of linear poly(dimethyl-co-hydromethyl)siloxanes (PDMHMS) with α-diketones. They only used a small amount of the catalyst, from 200 to 1000 ppm, and obtained silicone elastomers with similar properties to commercial ones cross-linked with platinum. However, the Si-O-C cross-link bonds made these elastomers less stable in water, especially in acidic conditions. The degradation rate could be controlled by changing the amount of Si-H in PDMHMS and by choosing α-diketones with a different steric bulkiness. This degradability could offer a new way to recycle these materials. They also reported a two-step method to add functional groups to a silicone elastomer by reacting PDMHMS with different monoketones in the presence of TPFPB, and then cross-linking it with α-diketone (Scheme 39) [121].

**Scheme 40:**
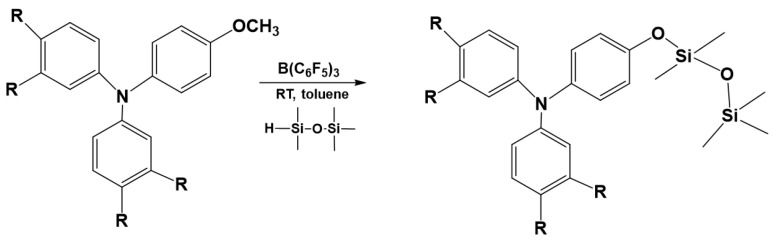
Synthesis of triarylamines with oligosiloxy functional groups from their methoxy derivatives using the P-R reaction.

The attachment of siloxane chain fragments to a large aromatic molecule can change its state from crystalline to liquid. This concept was explored by Brook and co-workers, who attached 1,1,1,3,3-pentamethyldisiloxane (MM^H^) or 1,1,1,3,5,5,5-heptamethyl trisiloxane (MD^H^M) to various aryl-methoxy-substituted triarylamines via the P-R reaction, producing several new liquid arylamines that could serve as novel components of optoelectronic devices and new organic semiconductors (Scheme 40). It is worth noting that the Lewis basicity of some aromatic triamines does not affect the catalytic activity of TPFPB and the P-R reaction occurs at room temperature at a relatively low concentration of 1% TPFPB. This can be explained by the formation of a weak one-electron transfer pair between TPFPB and the triarylamine, which is in equilibrium with the free borane [122,123].

A novel molecular liquid fluorescent ink was constructed using the P-R reaction of tetrakis(4-methoxyphenyl)ethylene (TPE) with Si-H-functionalized siloxane oligomers. The obtained siloxane-functionalized TPE is a low-viscosity liquid [124].

**Scheme 41:**
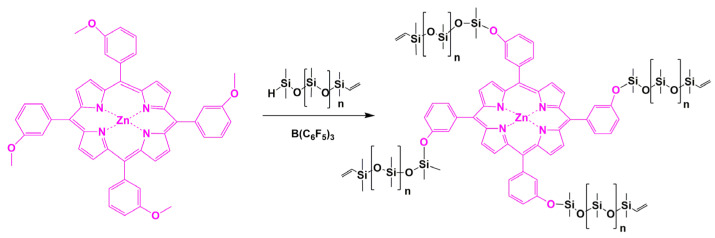
Functionalization of the porphyrine–Zn complex with oligosiloxane side groups.

The attachment of oligosiloxane chains to larger molecules makes them compatible with silicone elastomers. This approach was exploited by Brook and co-workers to link polysiloxanes via P-R condensation with a 5,10,15,20-(tetra-3-methoxyphenyl)porphyrine-Zn complex [125] (Scheme 41), which is a photosensitizer. This modified porphyrin is well dispersed in the silicone material and may form covalent cross-links in the silicone matrix using vinyl groups at siloxane chain ends. The photosensitizer enables the generation of singlet oxygen, ^1^O_2_, which is very active against bacteria. Coating medical devices with such a polymer prevents infections caused by the use of these devices.

Skov, in collaboration with Brook, used a porphyrin-Zn complex modified with bis(trimethylsiloxy)methylsilane using the P-R reaction as an additive to a silicone elastomer. They achieved a homogeneous incorporation of up to 10% of the modified porphyrin-Zn complex, which increased the actuations in response to an electric field by more than 40% [126]. Xin Dong Xu et al. later used the same reaction to make the porphyrin-Zn complex compatible with a luminescent silicone elastomer [127].

**Scheme 42:**
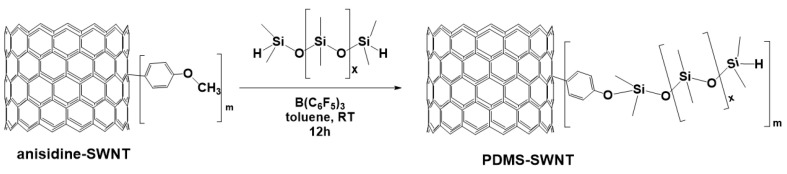
Modification of the carbon nanotubes with Si-H-terminated oligosiloxanes in P-R conditions.

Using the P-R reactions, single-walled carbon nanotubes were modified with SiH-terminated oligosiloxane. The nanotubes were first pre-grafted with p-anisidine to generate methoxyphenyl groups on their surface, which allows for this modification (Scheme 42). This modification strongly improved the dispersity of the nanotubes in organic solvents [128].

## 5. Hydride Transfer from Si to H: Dehydrogenative Condensation Leading to SiOSi

TPFPB is a very effective catalyst of the dehydrogenative condensation of hydrosilanes with silanols (Table 2, Row 3). The first application of this reaction to the polymerization and cross-linking of siloxane polymers was reported by Deforth and Mignani from Rhodia in 2003 [129]. Subsequently, Kawakami with co-workers used this reaction to obtain optically active phenylmethyl-substituted siloxane oligomers and copolymers [130]. The dehydrogenative condensation has found a wide application in the syntheses of polysiloxanes and siloxane copolymers. This type of polycondensation is also catalyzed by noble-metal complexes [6,7].

**Scheme 43:**
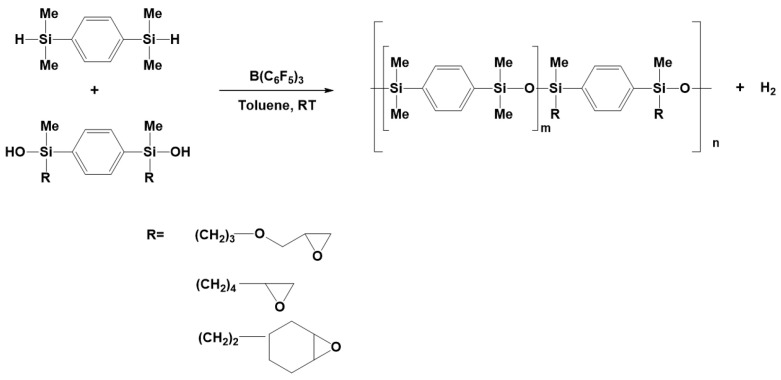
Synthesis of epoxy-functional poly(silphenylenesilyloxanes) with dehydrogenative condensation catalyzed by TPFPB.

Xue and Kawakami synthesized various linear poly(silphenylenesilyloxanes) including epoxy-functionalized copolymers using the condensation of 1,4-bis(hydroxydimethylsilyl)benzene or 1,4-bis(hydroxymethyl(epoxyalkyl)silyl)benzene with 1,4-bis(dimethylsilyl)benzene or a silphenylenesiloxane prepolymer terminated with SiH groups (Scheme 43) [131]. They found that TPFPB was a better catalyst for these reactions than di-n-butyltin dilaurate. They obtained siloxane copolymers with a molecular weight above 1 × 10^4^ and preserved epoxide groups in a high yield in the presence of TPFPB. This indicates that the dehydrogenative condensation is much faster than the reductive opening of the epoxide ring by the SiH group.

**Scheme 44:**
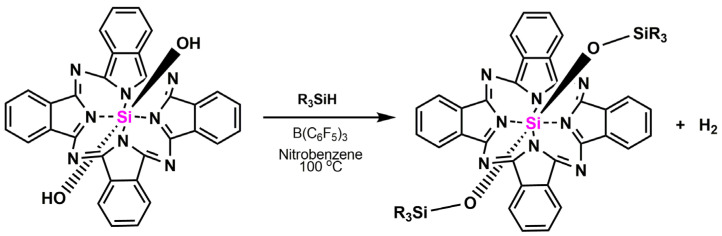
Functionalization of phthalocyanines with SiH-functional silanes using dehydrogenative condensation catalyzed by TPFPB.

The SiH + HOSi condensation promoted by TPFPB proved to be suitable for introducing organosilane or siloxane groups to large complex molecules that may be difficult or impossible to modify with other methods. A good example is the functionalization of phthalocyanines with SiH-functional organosilanes or siloxanes, which Bender et al. successfully carried out (Scheme 44) [132]. Phtalocyanines are widely used as dyes and pigments, and their modification with silane or siloxane groups may increase their solubility or dispersion ability in various polymers.

**Scheme 45:**
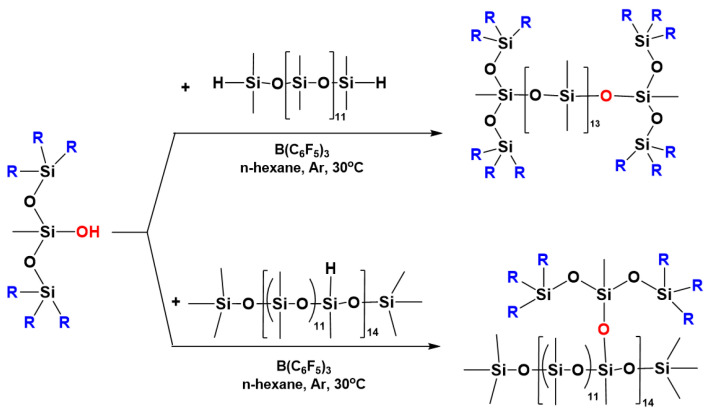
Synthesis of well-defined polysiloxanes using the dehydrogenative condensation of siloxanol-functional dendrons with SiH-functional polysiloxanes.

Arzumanyan and co-workers prepared well-defined polysiloxanes of various architectures, including dumbbell-shaped and bottlebrushed ones, using TPFPB-promoted SiH + SiOH condensations of linear polydimethylsiloxanes with a Si-H terminal or internal groups with siloxanol-functional dendrons [133]. Scheme 45 illustrates examples of these syntheses.

**Scheme 46:**
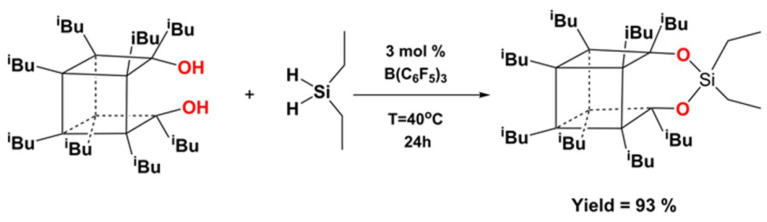
An example of the dehydrogenative coupling of POSS silanols with Si-H-functional compounds in the presence of TPFPB.

A highly selective method of the functionalizing of cage and opened-cage silsesquioxanes containing silanol groups (POSS silanols) with a siloxane group bearing various substituents was based on the dehydrogenative coupling of POSS silanols with hydrosilanes promoted by TPFPB [134]. The POSS silanols as well as disilanol and trisilanol of opened silsesquioxane cages underwent reactions with various organohydrosilanes. Diethylsilane was used to close the cage of opened silsesquioxane diol, as shown in Scheme 46.

**Scheme 47:**
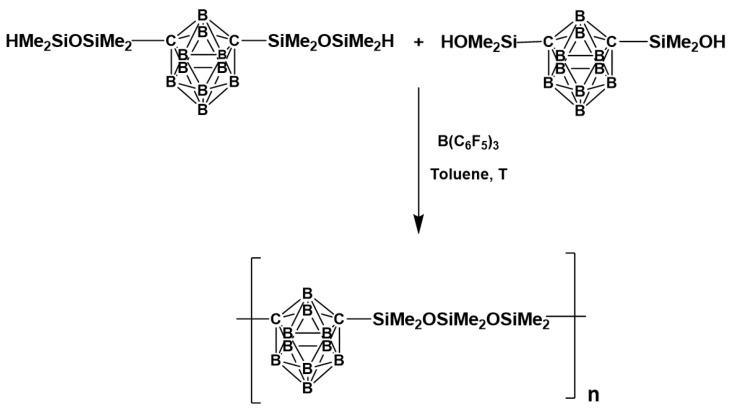
Synthesis of the polycarbosiloxane copolymers with dehydrogenative condensation.

TPFPB-catalyzed dehydrogenative condensation offers an easy route to polycarbosiloxane copolymers, which have been successfully obtained by reacting appropriate carboranes functionalized with siloxanediol and dihydrosiloxane groups (Scheme 47) [135].

**Scheme 48:**
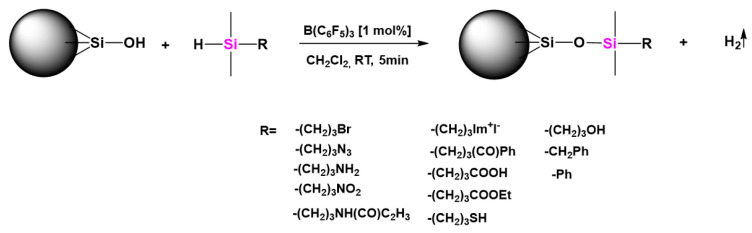
Rapid modification of the surface of amorphous silica particles with functional organosilanes.

Shimada et al. demonstrated the effective and rapid modification of the surface of amorphous silica by means of a dehydrogenative condensation reaction between silanol groups on its surface and organohydrosilanes containing various functional groups, such as halogens, azide, nitro, alcohol, ketone, thiols and others, at room temperature in the presence of 1 mol% TPFPB (Scheme 48). Interestingly, the authors claim that the functionalization of the silica surface with the basic 3-aminopropyldimethylsilane or highly reactive 3-hydroxypropyldimethylsilane was successful under these conditions [136].

Zhang et al. used a similar method to modify hydrophilic nanosilica with polymethylhydrosiloxane. The highly hydrophobic silica was prepared in a few minutes at room temperature and used as a reinforced filler in a styrene–butadiene rubber composite. The composite with this modified silica showed dramatic improvements in mechanical and dynamic properties such as wet traction and rolling resistance and is a promising material for use in green tires [137].

The rapid silanization of a hydroxyl-terminated porous silicon surface with dimethylphenylsilane in the presence of TPFPB was reported by Voelcker et al. [138]. The resulting hydrophobic porous silicon surface was resistant to hydrolytic etching in an aqueous environment.

Zuilhof and co-workers used this methodology to modify the native SiOx layer of a silicon wafer with organodimethylhydrosilanes, RMe_2_SiH, where R is the hydrocarbon chain from C_8_ to C_18_.The process was performed in methylene chloride containing 1% of TPFPB in 5 min at room temperature [139]. The obtained surface covered with an organosilane monolayer had a static water contact angle between 103° and 111°, indicating that the long-chain organohydrosilane forms a densely packed hydrocarbon layer. The silicon surface modified in this way showed an improved hydrolytic stability in acidic media and a physiological PBS solution. They extended this methodology to rapidly prepare a superhydrophobic surface on silicon nanowires by reacting with (perfluorooctyl)dimethylsilane under similar conditions.

**Scheme 49:**
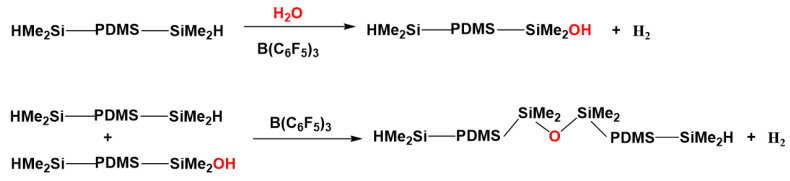
Synthesis of high-molecular-weight linear polysiloxane from Si-H-terminated siloxane oligomers in the presence of a controlled amount of water using the partial hydrolysis of Si-H groups and dehydrogenative condensation.

Brook and co-workers showed that organohydrosilanes and hydrosiloxanes dissolved in hydrocarbon solvents underwent hydrolysis to silanols in the presence of water and catalytic amounts of TPFPB. The formed silanols then underwent a rapid dehydrogenative condensation with the remaining SiH groups, forming a new siloxane bond. This behavior enabled the polycondensation of SiH-terminated siloxane oligomers as well as the polycondensation of simple R_2_SiH_2_ monomers with a controlled amount of water. They demonstrated the application of this strategy to the synthesis of high-molecular-weight linear polysiloxanes (Scheme 49) [140]. The resulting telechelic SiH or SiOH-terminated polymers had a polydispersity below two and a low content of cyclic oligomers. This synthetic strategy was also an easy route to siloxane block copolymers. Interestingly, tris(trimethylsiloxy)silane, M_3_T^H^, was unreactive under these conditions, probably for steric reasons.

Skov and co-workers synthesized macrocyclic polydimethylsiloxane by using a hydrolysis–dehydrogenative coupling sequence promoted by TPFPT of SiH-terminated PDMS. They applied high-dilution conditions to favor end to end ring closure reactions over the linear growth of macromolecules. They separated linear macromolecules from macrocyclic polydimethylsiloxane with the TPFPT-catalyzed condensation of SiH chain ends with silanol groups on silica gel [141].

## 6. Hydride Transfer from Si to H: Dehydrogenative Condensation Leading to SiOC

**Scheme 50:**
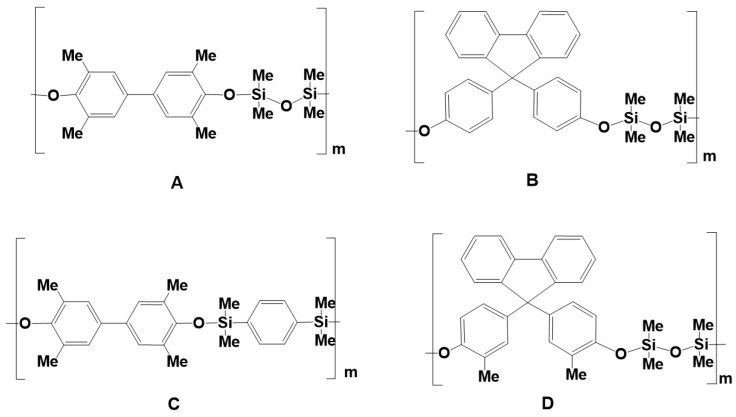
Examples of polyaryloxysiloxanes prepared with the dehydrogenative condensation: (**A**)—TMDS with TMBP; (**B**)—TMDS with BPF; (**C**)—BDMSB with TMBP; (**D**)—TMDS with DMBPF.

Cella and Rubinsztajn prepared series of polyaryloxy siloxanes and polyaryloxysilanes with the dehydrogenative polycondensation of organodihydrosilanes and siloxanes, such as 1,4-Bis-dimethylsilylbenzene (BDMSB)and 1,1,3,3-tetramethyldisiloxane (TMDS), with various bis-phenols, such as 3,3′,5,5′-tetramethylbiphenyl-4,4′-diol (TMBP), 4,4′-(9H-fluorene-9,9-diyl)diphenol (BPF), 4,4′-(9H-fluorene-9,9-diyl)bis(2-methylphenol) (DMBPF) [110]. Even hindered phenols and silanes readily entered this reaction. Examples of polymers obtained are shown in Scheme 50. Obtained polymers were hydrolytically and thermally resistant.

**Scheme 51:**
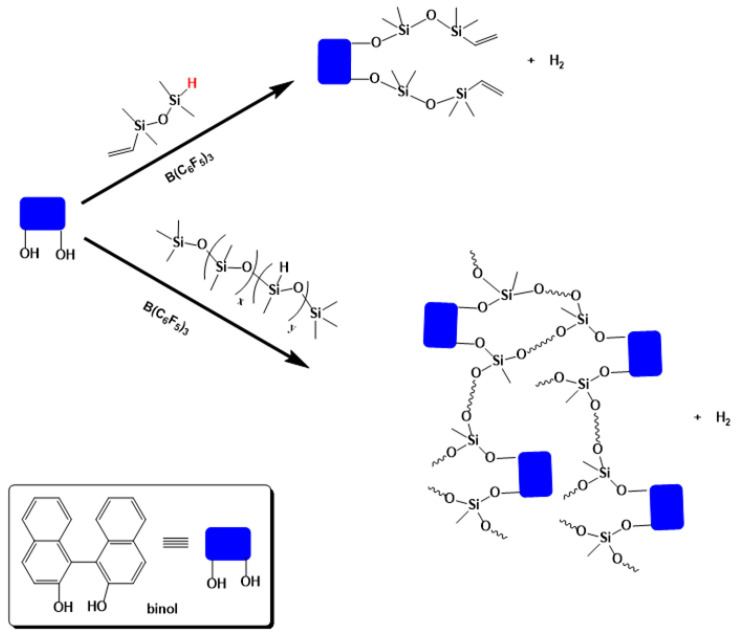
Dehydrogenative coupling of binol with SiH-terminated oligosiloxanes.

The dehydrocoupling of binaphthol (binol) with short-chain-SiH-terminated polysiloxanes resulted in a series of linear siloxane copolymers with binol units incorporated into their chains [142]. The binol units increased the refractive index of the polymer. The dehydrogenative condensation of binol with 1-vinyl-3-hydrotetramethyldisiloxane in the presence of TPFPB produced a divinyl-terminated macromere (Scheme 51). This macromer underwent a Pt(0)-catalyzed hydrosilylation-addition polymerization with diphenyldihydrosilane forming a linear siloxane binaphthol copolymer. The dehydrogenative condensation of binol with SiH groups can also be used for the cross-linking of linear poly(dimethyl-co-hydromethyl)siloxanes (PDMHMS), leading to the formation of silicone foams or transparent elastomers.

Bender and co-workers synthesized phenoxylated polysiloxanes by using dehydrogenative condensation promoted by TPFPB. They reacted linear poly(hydromethylsiloxane-co-dimethylsiloxane) with phenolic compounds such as phenol, 4-tert-octylphenol and 3-pentadecylphenol under P-R conditions [143]. The reaction with 3-pentadecylphenol, which was derived from naturally sourced cardanol, produced a waxy silicone-based solid.

**Scheme 52:**
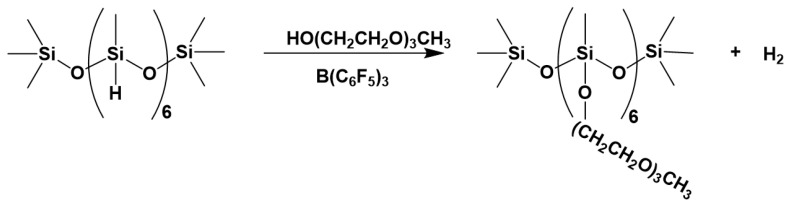
Synthesis of novel siloxanes functionalized with oligoethylene glycol side groups.

West et al. employed dehydrocarbonative condensation catalyzed by TPFPB to functionalize pentamethylcyclopentasiloxane (D^H^_5_) and linear siloxane oligomers (MD^H^_6_M) with oligoethylene glycol moieties (Scheme 52) [144]. The synthesized compounds were tested as novel solvent-free polymer electrolytes. They formed homogeneous amorphous complexes with lithium bis(trifluoromethanesulfonyl)imide and showed their higher conductivity than analogous compounds, where the oligoethylene glycol group is connected to the Si atom via trimethylene linkage.

Brook and co-workers showed that the solubilization of lignin originating from hard woods can be achieved under mild conditions using silylation with pentamethyldisiloxane (MD^H^) in the presence of TPFPB [145]. This silylation process involved SiH + HOC dehydrogenative condensation as well as the reduction of some organic groups and the degradation of the lignin network. The results of studies of reactions of MD^H^ with model lignin compounds suggested the mechanism of this silylation process. Lignin is the second most abundant natural polymer, so this process may provide an important source of new renewable materials.

Recently, the same group used an analogous synthetic approach to demonstrate the preparation of another silicone composite filled with a renewable material. They were able to mix a mixture of SiH-functional silicone fluids with 50 wt.% starch powder and catalytic amounts of TPFPB. The resulting mixture was converted into a cross-linked starch–silicone hybrid foam in a few minutes, using the dehydrogenating condensation of Si-H groups with alcohol groups present on the starch molecule [146].

**Scheme 53:**
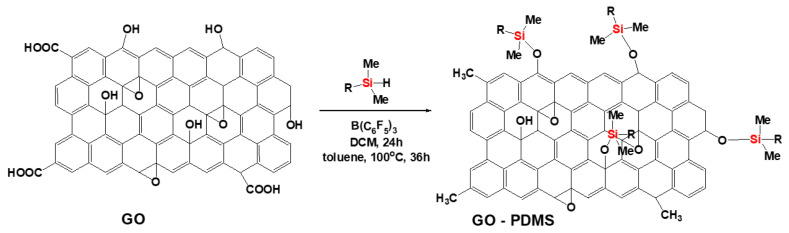
Modification of the graphene oxide (GO) surface with siloxane side groups.

Graphene is incompatible with polymers, which makes it difficult to incorporate this promising material into polymer matrices. This problem can be solved by attaching polysiloxane chains to its surface. For this purpose, the graphene surface was first oxidized to graphene oxide (GO) to generate hydroxyl groups, which were then subjected to dehydrogenative condensation with SiH-terminated polysiloxane in the presence of TPFPB (Scheme 53) [147].

## 7. Hydride Transfer from Si to Si

**Scheme 54:**
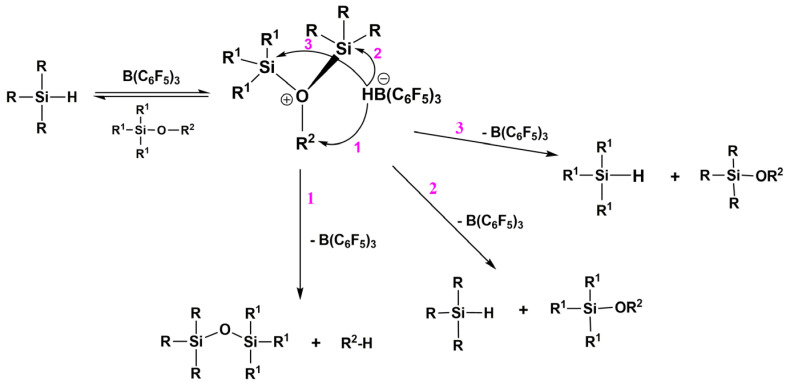
Mechanism of the P-R reaction showing three alternative pathways of this process. (1)—P-R reaction; (2)—back to substrates; (3)—metathesis.

Assuming that the oxonium ion paired with (C_6_F_5_)_3_BH^-^ is an intermediate in the P-R reaction, it can decompose in three directions, as shown in Scheme 54. The classic P-R reaction is a hydride ion to carbon transfer reaction (route “1”) that produces a hydrocarbon byproduct [37]. If the hydride ion follows path “2”, it returns to the parent silicon, restoring the starting compounds. On the other hand, the transfer of the hydride ion along path “3” causes a metathetic exchange of functional groups between the two silicon substrates.

**Scheme 55:**
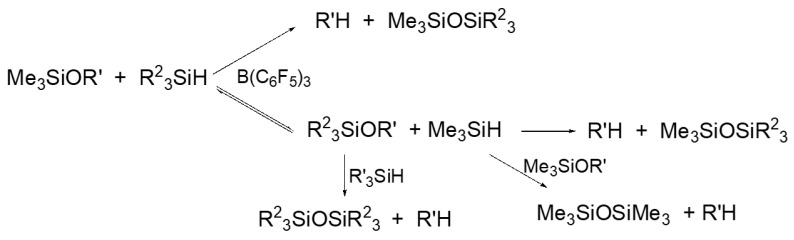
Reactions of model triorganoalkoxysilane with triorganosilane in the presence of TPFPB.

The P-R reaction, as opposed to the metathesis, is irreversible, creating a siloxane bond and a non-reactive hydrocarbon. In the process of metathesis, new reactive compounds are formed that can cross-react with substrates, as shown for the model system in Scheme 55.

This metathesis is usually an undesirable reaction that needs to be minimized, as it not only lowers the efficiency of the P-R process but also affects the structural control of the final product [95,96,102,148]. The structure of the reactants and the reaction conditions must influence the competition of the metathesis with the P-R reaction because, in many cases, the metathesis is eliminated and the P-R reaction enables a high level of the structural control of its products [22,47,63,65,77,78,97]. Kinetic studies have shown that isopropoxysilane reacted about a hundred times faster in P-R reactions than in the metathesis, but in many cases, the role of the metathesis in P-R reactions is significant [37]. Unfortunately, little is known about how the structure of the reactants affects the competition between metathesis and the P-R reaction. How do polar effects and steric substituents on silicon affect this competition? How do process conditions, such as the solvent and temperature, affect this competition? These questions need further research.

**Scheme 56:**
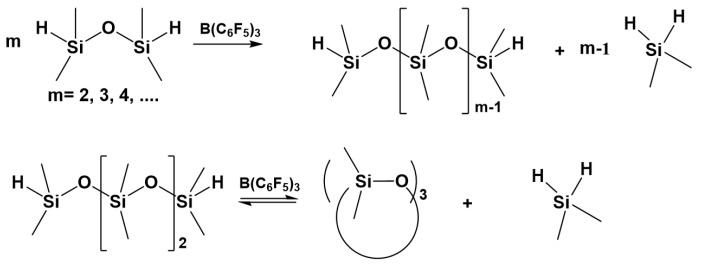
Inter- and intra-molecular metathesis of SiH-terminated oligosiloxanes in the presence of TPFPB.

The metathetic exchange can lead to interesting products when it occurs between two siloxane molecules containing OSiR_2_H end groups. For example, 1,3-dihydrotetramethyldisiloxane, HSiMe_2_OSiMe_2_H (^H^MM^H^), undergoes a rapid disproportionation (dismutation) involving the exchange of dimethylsiloxy units between its molecules in the presence of TPFPB [149,150]. This reaction yields oligodimethylsiloxanes terminated by the hydrodimethylsilyloxy groups, HSiMe_2_O(SiMe_2_O)_n_SiMe_2_H (^H^MD_n_M^H^) and dihydrodimethylsilane (Me_2_SiH_2_). Scheme 56 shows this process. The first step is a reaction between two ^H^MM^H^ molecules, leading to Me_2_SiH_2_ and 1,5-dihydrohexamethyltrisiloxane (^H^MDM^H^). Hexamethylcyclotrisiloxane, D_3_, is formed from 1,7-dihydrooctamethyltetrasiloxane (^H^MD_2_M^H^) in a reversible unimolecular reaction. All resulting products may have applications: Me_2_SiH_2_ is a monomer in CVD processes, SiH-terminated oligodimethylsiloxanes are a substrate for the synthesis of siloxane block copolymers and D_3_ is a monomer for the controlled synthesis of polydimethylsiloxanes with anionic ring-opening polymerization.

This disproportionation reaction may interfere with some other TPFPB-catalyzed hydrosiloxane reactions. Experimental and theoretical studies have shown that the hydrosilylation of some olefins with ^H^MM^H^ was overwhelmed by the metathetic formation of ^H^MDM^H^ trisiloxane [41]. A quasi-living polymerization of ^H^MM^H^ with a slow addition of water produced a significant amount of undesirable D_3_ [148].

**Scheme 57:**
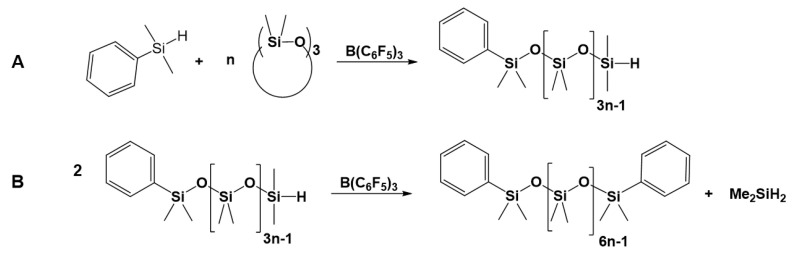
Hydride-transfer ring-opening polymerization of D_3_. **A**—chain propagation; **B**—termination via metathetic chain coupling.

A reaction that can be classified as a metathetic process involving H^-^ transfer is the hydride-transfer ring-opening polymerization (HTROP). It is a new type of cationic polymerization. It differs from the classic cationic polymerization initiated by Bronsted or Lewis acids, following a completely different mechanism. It requires initiation by a compound containing the Si-H functional group and the presence of TPFPB. Chain growth proceeds with hydride transfer from the chain end to the monomer through the mediation of TPFPB [151]. This polymerization occurs with strained cyclic siloxanes such as hexamethylcyclotrisiloxane, (D_3_). The polymerization of D_3_ initiated with about 12 mol% of HMe_2_SiOMe_2_H produced polydimethylsiloxane with HMe_2_SiO units at both chain ends, having the molecular mass Mn = 1.97 × 10^4^ with a polydispersity of 1.72. On the other hand, the polymerization of D_3_ initiated with about 8 mol% of monofunctional PhMe_2_SiH produced polydimethylsiloxane with PhMe_2_SiO units at both chain ends (molecular mass, Mn = 7600; polydispersity, 1.70). Chain propagation (Scheme 57A) is accompanied by the previously discussed process of dismutation between two siloxane molecules containing the OSiMe_2_H end group according to Scheme 57B. This process prevents the formation of the OSiMe_2_H-functionalized polymer at only one end of the chain.

The polymerization of D_3_ in the presence of TPFPB was also performed in an aqueous dispersion by Ganachaud and co-workers [152]. A high-molar-mass polymer was obtained (Mn = 6.3 × 10^4^, PD = 1.60). The mechanism considered by the authors is different from that of HTROP.

**Scheme 58:**
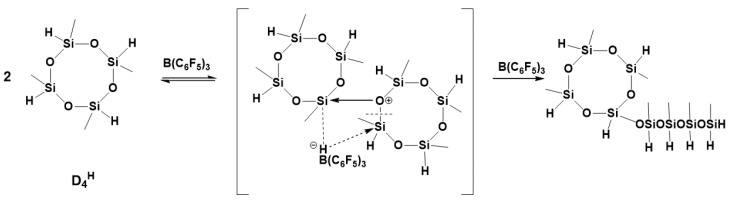
Initiation step of the hydride-transfer ring-opening polymerization of D^H^_4_.

**Scheme 59:**
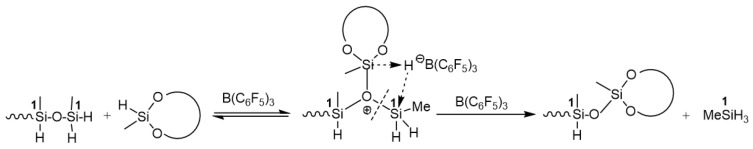
Termination step of the hydride-transfer ring-opening polymerization of D^H^_4_.

Particularly interesting is the HTROP of 1,3,5,7-tetramethylcyclotetrasiloxane (D^H^_4_) [153]. It proceeds with extensive branching, which is accompanied by an extensive intra-molecular cyclization, leading to a close multicyclic structure of the siloxane polymer formed. The HTROP of D^H^_4_ is a unique ring-opening polymerization of an unstrained cyclic monomer that leads to a complete monomer conversion and, when carried out in a dilute solution, can yield a soluble, highly branched polymer of a full cyclic structure with a molecular weight of 10^5^–10^6^ Daltons. There are no cyclic oligomers with a full monomer conversion. This process is initiated by hydride transfer between two monomer molecules, as shown in Scheme 58. The formed terminal unit, -OSiH_2_Me, is very reactive in chain propagation. Chain growth is completed with a hydride-transfer reaction from the monomer or polymer chain to this end group, forming a new siloxane bond and volatile MeSiH_3_, as shown in Scheme 59. The obtained polymer has no end groups, but many intra-molecular cyclic structures formed in the processes of initiation and termination and in the reaction of chain transfer to the polymer. This fully closed structure of the polymer makes it an interesting material, because many reactive side groups enable its easy functionalization and cross-linking.

**Scheme 60:**
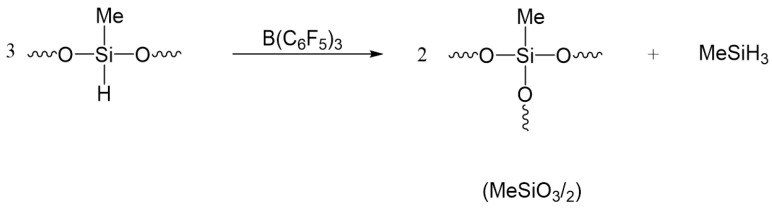
The general equation of the self-restructuration of PHMS in the presence of TPFPB.

Linear polyhydromethylsiloxane (PHMS) undergoes self-restructuring with analogous to HTROP hydride transfer between silicon atoms in the presence of TPFPB [154]. This process produces volatile MeSiH_3_, like the HTROP of D^H^_4_, and follows the general equation in Scheme 60. Quantum mechanical calculations confirmed the self-restructuring mechanism based on silicon to silicon hydride transfer.

The process can be carried out in a controlled manner in a dilute polymer solution at room temperature with a low catalyst concentration. The relatively short-chain linear PHMS (Mn ≈ 2000) in a 10% toluene solution converts easily to a high molecular weight hyperbranched polymer, Mw > 10^5^ Daltons. Polysiloxane thin films are produced with cross-linking PHMS at a high concentration. The cross-linking occurs as the solvent evaporates from the films, and the films are further cured in air at 150 °C.

## 8. Summary and Outlook

The Piers–Rubinsztajn (P-R) reaction is a novel way of creating siloxane bonds from alkoxysilanes and silane hydrides. The catalyst for this reaction is tris(pentafluorophenyl)borane (TPFPB), a strong Lewis acid that transfers a hydride anion from the silane to the carbon atom of the alkoxysilane, resulting in a formation of a new siloxane bond via dehydrocarbonative condensation. The only byproduct is a harmless, usually volatile, hydrocarbon. Rubinsztajn et al. discovered this reaction at General Electric Company in 2002 [19,20,21,22] based on earlier work by Piers et al. They described the use of this reaction for the synthesis of siloxane polymers and copolymers, hyperbranched polymers and the cross-linking of SiH-functional polymers under mild conditions in the presence of catalytic amounts of TPFPB. It was later found that the TPFPB also facilitates the transfer of a hydride anion from silicon to other elements like hydrogen and silicon, opening up new possibilities for creating novel materials that were previously inaccessible. The P-R reaction and related processes allow for a much better control of the structures of synthesized polysiloxanes than the processes used so far. Brook and their team recognized the value of these reactions early on and developed several methods to produce well-defined oligosiloxanes, linear siloxane copolymers, complex hyperbranched and dendritic polymers, 3D networks and even hybrid siloxanes with natural materials like soybean oil, lignin and starch. The P-R reactions were also applied to modify various surfaces, such as silica, carbon nanotubes and graphene oxide, by attaching SiH-functional organosilanes and siloxane polymers to them. The P-R reactions can be performed in the presence of many functional groups including alkenyl, haloalkyl, epoxide, thiol and even triarylamine and disilazane. The presented developments clearly show a large synthetic potential of these reactions regarding the preparation of novel well-defined siloxane materials for advanced applications such as electronic and photonic materials, biomaterials and ceramics.

However, to fully exploit the potential of the P-R reaction, further research is needed to improve the control of the selectivity of the dehydrocarbonative condensation with respect to metathetic functional group exchange; to reduce the sensitivity of this reaction to moisture, which can cause an unpredictable induction period; and to make the catalyst more resistant to degradation by SiH compounds. Although the general mechanism of the P-R reaction is known, further studies should be performed for a better understanding of the influence of the structure of reactants on the reactivity and selectivity of this process. Addressing these issues could lead to a wider acceptance and use of the P-R reaction within academia and the silicone industry.

## Figures and Tables

**Table 1:** Condensation reactions leading to the creation of siloxane bonds.

Reagents	Products
≡Si-X + HO-Si≡; where X = halogen	≡Si-O-Si≡ + HX
≡Si-X + RO-Si≡; where X = halogen	≡Si-O-Si≡ + RX
≡Si-X + RC(O)O-Si≡; where X = halogen	≡Si-O-Si≡ + RC(O)X
≡Si-X + MtO-Si≡; where X = halogen, Mt = Li, Na, K	≡Si-O-Si≡ + MtX
≡Si-H + HO-Si≡	≡Si-O-Si≡ + H_2_
≡Si-OR + HO-Si≡	≡Si-O-Si≡ + ROH
≡Si-O(O)CR + HO-Si≡	≡Si-O-Si≡ + RC(O)OH
≡Si-O(O)CR + RO-Si≡	≡Si-O-Si≡ + RC(O)OR
≡Si-NH_2_ + HO-Si≡	≡Si-O-Si≡ + NH_3_
≡Si-NR_2_ + HO-Si≡	≡Si-O-Si≡ + HNR_2_
≡Si-O(O)CCH_3_ + MtO-Si≡; where Mt = Li, Na, K	≡Si-O-Si≡ + CH_3_C(O)OMt

**Table 2:** Hydride transfer reactions from hydrosilanes (Si-H) catalyzed by tris(pentafluorphenyl)borane.

	Substrates	Products	Hydride Transfer
1	R_3_SiH + R′_3_SiOR″	R_3_SiOSiR′_3_ + R″H	From Si to C
2	R_3_SiH + R′OR″	R_3_SiOR′ + R″H	From Si to C
3	R_3_SiH + HOSiR′_3_	R_3_SiOSiR′_3_ + H_2_	From Si to H
4	R_3_SiH + HOR′	R_3_SiOR′ + H_2_	From Si to H
5	R_3_SiH + HOH	R_3_SiOH + H_2_	From Si to H
6	R_3_Si′H + R′_3_SiOR″	R_3_Si′OR″ + R′_3_SiH	From Si to Si
7	R_2_HSiOSiHR_2_ + R_2_HSi′OSi′HR_2_	R_2_HSiOSi′HR_2_ + R_2_HSiOSi′HR_2_	From Si to Si
8	R_2_HSiOSiHR_2_ + R_2_HSi′OSi′HR_2_	R_2_HSiOSiR_2_OSi′HR_2_ + R_2_Si′H_2_	From Si to Si
9	R_2_HSiOSiHROSiHR_2_ + R_3_Si′OSi′RH_2_	R_2_HSiOSi(OSi′R_3_)OSiHR_2_ + RSi′H_3_	From Si to Si

## Data Availability

Not applicable.
